# SnpHub: an easy-to-set-up web server framework for exploring large-scale genomic variation data in the post-genomic era with applications in wheat

**DOI:** 10.1093/gigascience/giaa060

**Published:** 2020-06-05

**Authors:** Wenxi Wang, Zihao Wang, Xintong Li, Zhongfu Ni, Zhaorong Hu, Mingming Xin, Huiru Peng, Yingyin Yao, Qixin Sun, Weilong Guo

**Affiliations:** Key Laboratory of Crop Heterosis and Utilization, State Key Laboratory for Agrobiotechnology, Beijing Key Laboratory of Crop Genetic Improvement, China Agricultural University, Beijing 100193, China

**Keywords:** SNP, database, server-framework, R/Shiny, wheat

## Abstract

**Background:**

The cost of high-throughput sequencing is rapidly decreasing, allowing researchers to investigate genomic variations across hundreds or even thousands of samples in the post-genomic era. The management and exploration of these large-scale genomic variation data require programming skills. The public genotype querying databases of many species are usually centralized and implemented independently, making them difficult to update with new data over time. Currently, there is a lack of a widely used framework for setting up user-friendly web servers to explore new genomic variation data in diverse species.

**Results:**

Here, we present SnpHub, a Shiny/R-based server framework for retrieving, analysing, and visualizing large-scale genomic variation data that can be easily set up on any Linux server. After a pre-building process based on the provided VCF files and genome annotation files, the local server allows users to interactively access single-nucleotide polymorphisms and small insertions/deletions with annotation information by locus or gene and to define sample sets through a web page. Users can freely analyse and visualize genomic variations in heatmaps, phylogenetic trees, haplotype networks, or geographical maps. Sample-specific sequences can be accessed as replaced by detected sequence variations.

**Conclusions:**

SnpHub can be applied to any species, and we build up a SnpHub portal website for wheat and its progenitors based on published data in recent studies. SnpHub and its tutorial are available at http://guoweilong.github.io/SnpHub/. The wheat-SnpHub-portal website can be accessed at http://wheat.cau.edu.cn/Wheat_SnpHub_Portal/.

## Introduction

Competition in the field of high-throughput sequencing greatly contributes to the reduction of sequencing costs. At present US $1,000 is the cost of sequencing ∼5 human genomes, 1 hexaploid wheat genome, 6 maize genomes, or 50 rice genomes at an average depth of 10×. Whole-genome sequencing is commonly used for species with mid-sized genomes such as soybean [1] and maize [2]. Genotyping-by-sequencing (GBS) or whole-exome-capture (WEC) technologies are also frequently used for large-genome species, such as wheat [3]. Currently, many wheat genome studies profile genomic variations on a scale of hundreds or thousands of accessions through WEC [4, 5] or whole-genome re-sequencing (WGS) [6].

The plant sciences have experienced a dramatic increase in the available genomic variation data due to the assessment of diverse species and plentiful germplasm resources. Beyond investigating the genetic diversity among individuals, large panels of high-quality genomic variation data have provided valuable resources and great opportunities for identifying trait-related genes, designing markers, constructing gene trees, exploring the evolutionary history, and assisting design of molecular breeding. Low-depth re-sequencing data from recombinant inbred line populations can assist in the identification of quantitative trait loci for traits of interest. Profiling the genomic variation of TILLING populations in crop studies can benefit the exploration of candidate variations that are rare in nature. The reuse of genomic variation data plays an important role in driving current plant science research.

As a routing pipeline, the raw reads obtained in whole-genome sequencing are first aligned to reference genomes. Then, single-nucleotide polymorphisms (SNPs) and small insertions/deletions (INDELs) are called and stored in standard VCF files [7]. There are great numbers of command line tools for bioinformaticians to manage and process VCF files. However, these files are usually quite large. The efficient management of the massive accumulated genomic sequencing data and exploration of these large-scale genomic variation data require computational skills exceeding the abilities of most biologists.

Some public databases are available for querying sample-specific genomic variations, such as the Information Commons for Rice (IC4R) database for rice studies [8] and MaizeGDB for maize studies [9]. These public databases are usually based on re-sequencing data that are generated and maintained by large international consortia. The web servers are implemented independently, providing different functions in exploring the genomic variations. With the increasing number of researchers generating new data worldwide, it has become impossible to maintain a centralized database that is both comprehensive and timely updated. There is great demand for implementing a universal platform for building distributed or private web servers for genomic data querying.

Several attempts have been made to implement web application frameworks. SNP-Seek II creates HDF files for storing genotypic data and uses Java Spring and ZK frameworks for implementing the web application architecture [10]. SNP-Seek II mainly supports data retrieval but is mainly designed for rice studies, and maintaining the complex computing structure requires professional technicians. CanvasDB is designed as a local database infrastructure for managing and retrieving the variation data using the MySQL database and supports filtering functionality and variation detection using R functions [11]. Gigwa v2 also imports VCF files in the NoSQL database, providing both analysis and visualization functions [12]. However, because relational databases are designed for table-structured data, systems such as MySQL are not the optimal method for managing complex genomic variation information, and uncompressed genomic variation data usually require a large amount of memory. SNiPlay3 is based on the Galaxy framework and provides a panel analysis that mainly focuses on whole-genome studies [13]. However, with the rapid accumulation of self-organized genomic variation data, there are still gaps in meeting the great requirements for a uniform, user-friendly, powerful web server framework with fast and efficient access to massive genomic variation data both locally and in a centralized location, to allow biologists to investigate genomic variations without the need for programming skills.

Here, we developed SnpHub as a uniform web server framework that can be easily set up on local server, can be applied by researchers for convenient management of massive processed VCF files, can be used to interactively explore genomic diversity and can be used to rapidly perform analyses in their own laboratories. SnpHub is designed for rapidly accessing SNP/INDEL data from specific regions and specific sample groups rather than performing whole-genome analysis. This framework is designed to be species independent, to support scalable variation data, and to provide resourceful and extendable functions for reusing and reanalysing genomic variation data.

## Methods

### The general SnpHub framework

**Figure 1:**
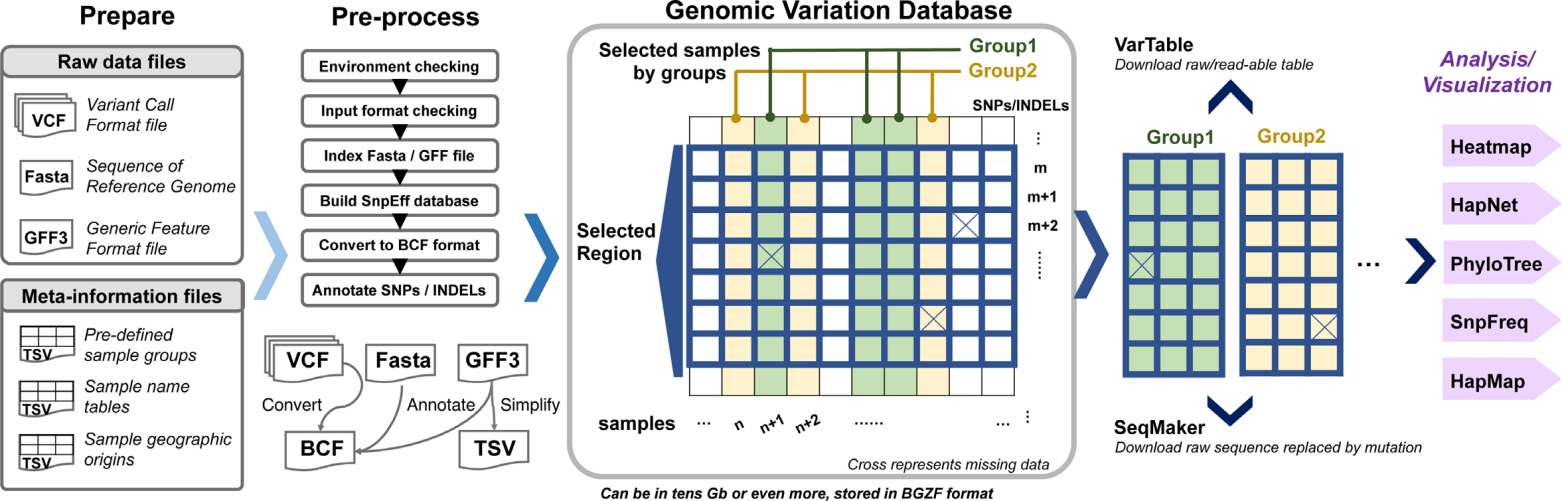
Design schema of the SnpHub server. Once the files and information tables are provided as indicated in the “Prepare” step, the SnpHub server instance performs a pre-processing step for building basic database files and then runs through the Shiny framework. Users can query specific genomic regions or genes for either pre-defined or customized sample groups. SnpHub can efficiently load the raw query data from the hard disk to RAM and then perform efficient analysis and visualization interactively.

The SnpHub framework is designed to be installed in the Linux system, using the Shiny/R framework and integrating several widely used bioinformatics command line software packages and R packages for analysing and processing genotyping data. SnpHub can be efficiently hosted on a modest computing server, with a local server installed with R-studio. Rather than performing a whole-genome general analysis, SnpHub provides an efficient way to quickly access data in a local region, filter sites and samples, and generate a genotype table as the intermediate data. To enhance the performance of reusing SNP/INDEL data for in-depth exploration, the intermediate genotype table is stored in RAM and then used for subsequent analyses and visualizations (Fig.   1).

The interactive user interface is implemented using the R/Shiny framework, with powerful, convenient functions for post-processing and visualizing the genotyping data. Considering that a large proportion of open-source bioinformatics analysis tools are implemented using R, SnpHub utilizes the R/Shiny framework to make it compatible and extendable. To simplify installation, a wrapped version for deploying the SnpHub Docker container is provided 14].

### Prerequisites for the set-up of SnpHub

#### Prepare step

The SnpHub server framework is designed to be lightweight and to rapidly access query information from the massive data stored on hard disks while requiring very little RAM. A general Linux workstation (for example, 4G RAM and 2.3 GHz dual process) installed with Shiny/R is enough to set up an instance of SnpHub. Several widely used bioinformatics software programs must be pre-installed, such as SAMtools [15], bcftools [16], seqkit [17], and Tabix [18], along with several R packages, such as ggplot2 [19], ggmap [20], dplyr [21], rjson [22], pegas [23], vcfR [24], ape [25], and DT [26].

To build a local instance, the VCF files, reference genome sequence file (FASTA format), gene annotation file (gff3 format), and metadata files defining sample information (TSV format) are needed. Providing meta-information such as sample information and pre-defined sample groups will enhance performance. A configuration template is provided for presenting meta-information such as the species name, sample description, reference genome, alignment method, and source of the accessions.

#### Pre-processing step

A shell wrapper program is provided for the pre-building process. When an instance server is built, users can access the data through a web browser interactively. Once the configuration information is provided, the local SnpHub instance can be easily built by running the shell wrapper in single command line. SnpHub will check the system environment for essential softwares and the formats of provided files. Then, the gene-based annotation of SNPs/INDELs will be performed by SnpEff [27]. All the meta-information is stored as tables on the hard disks, which is achieved by Tabix [18] for fast retrieval of the content.

## Key Features for Improving the Performance of SnpHub

### Rapid retrieval of genotype matrix by randomly accessing the disk

Considering that a relational database such as the mySQL framework is suitable for tables and requires a large amount of memory, SnpHub instead adopts the bioinformatician-friendly BCF format for storing the massive genomic variation data. BCF is a binary file format corresponding to VCF [16] with improved performance for supporting the fast querying of a subset of data by randomly accessing the hard disk, taking advantage of the BGZF compression format. In the pre-processing step, all the VCF files will be converted to BCF files. Another benefit is that bioinformaticians can directly perform analysis on these BCF files without storing another copy or format for the same dataset. To improve performance, SnpHub only retrieves a small piece of data for the selected region and selected samples from the disk instantly and stores the intermediate SNP/INDEL table in RAM to be efficiently processed by the downstream analysis functions.

### The triple-name strategy balances convenience and efficiency

To balance the requirements of convenience in management by server managers, ease of querying, and readability of the analysis result, SnpHub uses a triple-name/ID for a sample, which includes (i) the vcf-ID, (ii) the accession-name, and (iii) the display-name. The vcf-ID is a string name that is the same as that provided in the VCF files, avoiding the modification of the original VCF files. The accession-name is usually a short name, such as “S01, S02, S03,” so that it can be easily typed in the input box for querying a list of samples. The display-name is designed as a readable name to be displayed in the results and figures so that researchers can conveniently interpret the result. Arbitrary sample information such as sample passport or sample notes can be provided in additional columns. Once the SnpHub instance is set up, a sample information web page with a search engine is provided for navigating the names of the available samples and corresponding meta-information.

### Analysis with defined sample groups

A new feature of SnpHub is that it allows the querying of samples by groups, either using a pre-defined group-ID or defining new groups. When setting up the server instance, the database manager can define the system-wide group-IDs by configuring the TSV file. Then, the users can conveniently use group-IDs for querying a list of genes such as #group-ID. Also, the user can define a custom groupID for a list of samples with syntax in the following format: NewGroupA{Sample1, Sample2, Sample3}. With the defined groups, it will be convenient to refer to a list of samples using a group-ID instead of the full list of sample names. By default, SnpHub reserves the group-ID “#ALL” for querying all the samples in the cohort.

### Exporting the tables and figures

SnpHub allows users to export tables in CSV format. Beyond interactive visualization of data by the many analysis tools, all the figures can be exported in both PNG and PDF formats. The exported PDF figure represents the vector graphic, as users can conveniently post-edit the figures using tools such as Illustrator. A panel of parameters is provided for formulating the height and width of exported figures to produce a satisfactory layout. To be reproducible and traceable, the time stamp and main parameters are appended to the exported figures.

## Results

### Main functions provided by SnpHub

**Figure 2:**
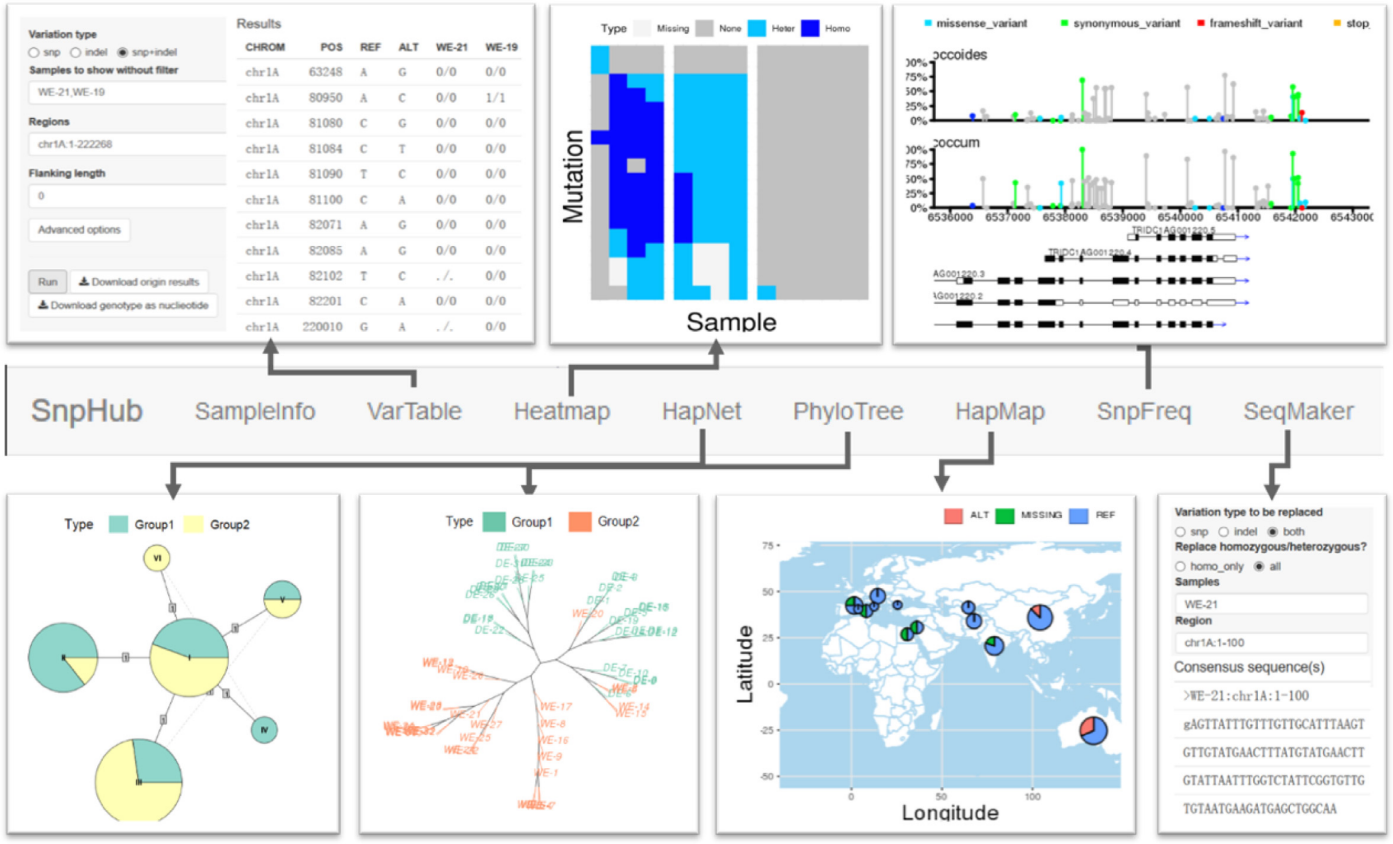
Analysis and visualization functions of the SnpHub server. In an SnpHub instance, each function is implemented in an independent web page tab.

SnpHub supports the navigation of massive genomic variation data by users by specifying a list of samples and specific genomic regions and performing lightweight analyses and visualizations through the Shiny/R framework. Uniform, flexible interfaces for manipulating the query parameters are provided. Because many open-source bioinformatics tools are implemented as command lines or R packages, the Shiny/R framework could be extended for integrating new tools for processing genomic variation tables. SnpHub provides user-friendly functions for navigating genomic variation data by implementing each of the functions on an independent tab page (Fig. 2). Raw variation data and genomic sequence retrieval functions are provided in VarTable and SeqMaker. Versatile analysis and visualization functions are provided, including Heatmap, HapNet, PhyloTree, SnpFreq, and HapMap. In all of these functions, SnpHub directly queries a gene ID as the corresponding genomic region directly based on the provided GFF file.

### VarTable, for exporting region-specific variation tables

In the VarTable function, users can query gene-/region-specific SNP/INDEL tables for a list of samples. To be consistent with the VCF format, the exported genotypes are denoted as “0/0”, “1/1”, “0/1” or “./.”, representing the “same genotype with reference genome,” “homozygous variant genotype,” “heterozygous genotype,” or “missing data,” respectively. Tables can be downloaded as raw data or as specific genotypes. This function supports a panel of parameters for filtering sites, such as the minimum allele frequency (MAF) and the maximum missing data frequency. To specify a region, users can either provide a specific region such as “chr:startPos-endPos” or use a gene-ID together with a parameter for the length of the flanking region. To support different purposes for discovering interesting variations, SnpHub extends the sample-based filtering interface to 3 categories: (i) samples must exhibit genotype variations compared with the reference genome; (ii) samples must be consistent with the reference genome in their genotype; and (iii) samples shall be exported in parallel without filtering on the basis of genotypes. Beyond the genotype, the metadata that are stored in VCF format, such as the read depth (DP), genotype quality (GQ), and variant annotations generated by SnpEff (ANN), can also be exported when the optional parameter boxes are checked.

### Heatmap, for visualizing genotypes in a matrix

The Heatmap function is an intuitive way to visualize tabular genotype information as a heat map graph. The samples to be visualized can be provided in 1 group or a list of groups. By default, genomic positions are displayed along rows, and samples are displayed in columns. The parameters of the 2 dimensions can be exchanged. Different colours are used to represent homozygous mutations, heterozygous mutations, reference genotypes, and missing data. To be more intuitively for visualizing haplotypes, samples are clustered within each group according to their genotype similarity. This function can be useful for exploring group-specific haplotypes or genotype patterns.

### HapNet, for constructing a haplotype network

The HapNet function provides an interface for constructing a haplotype network, which is widely used for characterizing the relationships among population based on sequences. The R package pegas [23] is used for generating the median-joining haplotype network plots. In the HapNet plot, each node represents a haplotype, whose radius is proportional to the number of samples harbouring this haplotype. The distance matrix is calculated among haplotypes based on their sequence distances. Finally, a minimum spanning tree is constructed. If multiple groups are provided, the nodes will be extended to a pie chart showing the proportion of each group. Similar haplotypes are joined by edges, with the distance shown on the edges. The haplotype network is usually used for exploring the evolutionary paths of different haplotypes among groups of samples [28].

### PhyloTree, for visualizing sample distance in a local region

The PhyloTree function supports the exploration of the gene-based genetic distances and evolutionary history based on high-density SNP data. The distance matrix is calculated on the basis of the genetic distances between accessions for specified genomic region. Then, 2 distance-based clustering methods, neighbour-joining (NJ) tree analysis and multidimensional scaling (also known as principal coordinates analysis), are supported. NJ-tree analysis can rapidly evaluate a large amount of data and is suitable for exploring the genetic relationships among samples for a specific region with a low time cost. Versatile layouts for visualizing the NJ-trees are available, including phylogram, cladogram, unrooted, fan, and radial layouts. Samples in different groups are shown in different colours. The multidimensional scaling analysis supports the visualization of the distances of samples in 2 dimensions through non-linear dimensionality reduction. This function provides users with multiple ways to visualize the sample distances for a local region.

### SnpFreq, for visualizing the SNP annotation in lollipop format

The SnpFreq function allows users to visualize the SNPs/INDELs and functional annotations along with the transcript-tracks. The previously proposed Lollipop graph [29] is adopted to visualize the positions and frequencies of genomic variations to distinguish the low-frequency variants and undetected variations. Variants are annotated in different colours, including missense variants, synonymous variants, frameshift variants, stop code gained/lost, and splice region variants. Transcripts in the same region are displayed as different tracks at the bottom, indicating the exons, introns, coding sequences, and transcription directions. Samples in different groups are summarized independently and visualized in different tracks, which can be useful for exploring the different frequencies of SNPs between groups.

### HapMap, for visualizing the genotypes geographically

The HapMap function provides a way to project the allele distribution of a single site geographically on a map, using the provided resource-gathering locations. A specific genomic site is required for the querying input boxes, such as “chr:pos.” If a genomic region is provided, the first variant site in this region will be used for the analysis. To increase user-friendliness, this function allows users to adjust the ranges of both longitudes and latitudes. A parameter is provided for the user to select the proper distance for merging geographically closely distributed accessions in 1 circle. This function could help to shed light on the spreading paths or histories of certain genomic variations/haplotypes.

### SeqMaker, for creating consensus sequence for an individual

The SeqMaker function can help to create a consensus sequence by substituting variants based on the reference genome, and the result can be downloaded directly as a FASTA file. In principle, this function retrieves a sample-specific sequence by replacing the detected genomic variations, which could be useful for sequence comparisons or primer design. However, it should be noted that the consensus sequences may not reflect the real sequences, considering the missing data as a result of sequencing coverages. Additionally, large structural variants are usually difficult to be detected by re-sequencing. By default, “#RAW” is preserved for retrieving the raw sequence in the reference genome.

## Advantages of SnpHub in Managing Variation Data

SnpHub is designed as a database framework specialized for retrieval and lightweight analysis of large genomic variation data. To provide instant responses for queries and interactive analysis, SnpHub focuses on the supports for haplotype analysis or genomic variation analysis for a specific region or gene, rather than genome-wide scale analysis such as genome-wide association studies analysis. For a clear view on the advantages of SnpHub, a comprehensive comparison on their supported features is presented (Table 1) with 3 other popular frameworks. Both Gigwa v2 [12] and CanvasDB [11] are specialized frameworks for investigating genotype data and are implemented with SQL-based database engines. The SQL-based servers generally require genotype information to be reloaded into specialized database tables, and meanwhile the useful meta-information in VCF files for describing variations is lost. SnpHub is actually based on BCF format, which is a lossless binary converted format of VCF and widely used by bioinformaticians, and thus will save disk storage in practice. JBrowse [30] is a general-purpose genome browser framework and provides flexible visualization and querying functions, although it has shortcomings in support of reanalyses. In contrast, SnpHub is designed to provide a variety of functions for both visualization and reanalysis, based on the R/Shiny framework. Moreover, as R packages and the R/Shiny framework are widely accepted by the bioinformatics community, it would be easier for SnpHub to incorporate powerful analysis functions and be extended. In general, SnpHub allows users an alternative choice for interactively exploring huge genomic diversity datasets and is stronger in performing lightweight reanalyses, including group-wise comparison, haplotype-related analysis, phylogenetic analysis, passport visualization, retrieving consensus sequences, and generating processable tables and figures.

**Table 1:** Features supported by SnpHub, Gigwa v2, CanvasDB, and JBrowse

Feature	SnpHub	Gigwa v2	CanvasDB	JBrowse
General design	Specialized	Specialized	Specialized	Generalized
Main strengths in querying data	Query with support for export, visualization, and reanalysis	Query with API for external visualization	Query with filtering functions	Track-based query and visualization
Database implementation	Indexed BCF	MongoDB	MySQL	Indexed VCF
Programming language	R/Shell	Java/JavaScript	R	JavaScript/Perl
Support downstream haplotype analyze	Yes	No	No	No
Allow sample selection	Yes	Yes	Yes	No
Forms of results	Table and Figure	Table	Table	Track-based plot
Support user-defined groups	No limitation in group number	≤2 groups	No	No
Deployment difficulty for bioinformaticians	Easy	Hard	Hard	Moderate
Visualizing variations across gene structure	Yes	No	No	Yes
Visualizing samples passports geographically	Yes	No	No	No
Access to metadata	Yes, user readable	Yes, built-in	No	Yes, user readable
Accession name management strategy	Triple-name strategy	Not provided	Not provided	Not provided
Retrieval consensus sequence	Yes	No	No	No

## Construction of the Wheat-SnpHub-Portal by SnpHub

Bread wheat is one of the most important staple crops and exhibits a large, repetitive genome whose genome size is estimated to be ∼16 Gb. As a hexaploid plant, bread wheat has a complex polyploidization history [31]. Following the release of high-quality reference genomes of wheat and its progenitors, a number of population genomics studies were released together with raw sequencing data or genomic variation data. Jordan et al. sequenced 62 lines of bread wheat (AABBDD) using WEC and GBS methods [32]. Two large WEC-based wheat population genomic studies sequenced 1,026 lines [4] and 487 lines [5]. Recently, Cheng et al. performed a high-resolution resequencing study of 93 hexaploidy wheat lines [6]. Population genomics data of wheat progenitors are also available, including data for wild and domesticated emmers (AABB) [33, 34] and of *Aegilopstauschii* (DD) [35].

Using all the above published datasets (Table 2), we constructed the “Wheat-SnpHub-Portal” website. The VCF files from He et al. [4] and Pont et al. [5] were downloaded from the links provided in their original articles. For datasets from Cheng et al. [6] and Wang et al. [33], the genotyping data in VCF format were shared by the authors. For the dataset from Singh et al. [35], raw sequence reads were downloaded from NCBI SRA under accession SRP141206 and VCF files were regenerated using scripts provided in the article. As for the datasets of Jordan et al. [32] and Avni et al. [34], we downloaded raw sequence data from NCBI SRA under SRP167848 and SRP032974, respectively. Raw reads were then trimmed using Trimmomatic [36] and aligned to reference genomes using BWA-MEM [37]. SNPs and INDELs were identified with the HaplotypeCaller module of GATK [38] and were further filtered by VariantFiltration function with the parameters “QD<2.0 || FS>60.0 || MQRankSum<−12.5 || ReadPosRankSum<− 8.0 || SOR>3.0 || MQ< 40.0 || DP >30 || DP < 3.” and “QD< 2.0 || FS>200.0 || ReadPosRankSum<−20.0 || DP>30 || DP< 3,” respectively. Generally, with the provided configuration data and variation files, the pre-processing step can be quickly finished, taking from ∼8 minutes [5] to ∼4 hours [6].

**Table 2:** The SnpHub instances available in Wheat-SnpHub-Portal

Ploidy	Method	No. Sample	Disk usage	Source
Tetraploid	WGS	35	15.0 GB	Wang et al. 2020 [33]
Hexa-/Tetra-/Diploid	WGS	63/25/5	39.8 GB	Cheng et al. 2019 [6]
Hexa-/Tetra-/Diploid	WEC	436/38/13	192 MB	Pont et al. 2019 [5]
Hexaploid	WEC	1,026	1.8 GB	He et al. 2019 [4]
Hexaploid	WEC and GBS	62	2.4 GB*	Jordan et al. 2015 [32]
Tetraploid	WEC	64	645 MB	Avni et al. 2017 [34]
Diploid	GBS	567	234 MB*	Singh et al. 2019 [35]

* Data are reanalysed from raw sequencing data. GBS: genotyping-by-sequencing; WEC: whole-exome-capture; WGS: whole-genome resequencing. Disk usage refers to the size of BCF files.

The Wheat-SnpHub-Portal website is designed as a portal website by providing multiple querying servers of variation databases for wheat and its progenitor species. Researchers studying wheat or wheat progenitors can easily explore multiple genomic variation datasets, as supported by the SnpHub framework. The Wheat-SnpHub-Portal website will be updated with further released genomic variation datasets of wheat and its progenitors in the future.

## Discussion

With decreasing sequencing costs, increased numbers of samples and species will be sequenced. That will make it difficult for universal and centralized databases to satisfy the versatile needs for variant analysis and querying new datasets. SnpHub can be applied to any species with an assembled genome and gene annotations. It can be instantly set up based on the VCF files. For future population genetic studies, a SnpHub querying server can be easily set up in addition to the publication of the raw data generated, making the data more easily accessible by the community. SnpHub can serve as a laboratory-level web server for navigating and visualizing genomic diversity or an individual line or lineage. SnpHub can be useful for different occasions: investigators can infer trait-associated genes with population structure information and variation function annotations from specific sample sets; and breeders can access the genetic diversity at specific loci for designing new breeds. SnpHub provides a uniform server framework for easily setting up distributed servers for genotyping queries and analysis and can be used to build database portals such as our Wheat-SnpHub-Portal, extending this strategy from wheat to other important crops or other plants.

## Availability of Supporting Source Code and Requirements

Project name: SnpHub

Project home page: https://guoweilong.github.io/SnpHub/

RRID:SCR_018177; biotoolsID: SnpHub

Operating system(s): Linux

Programming language: R, Shell

Other requirements: R/Shiny, samtools, bcftools, seqkit, tabix

License: MIT licence

An archival copy of the GitHub repository is available via the *GigaScience* database GigaDB [39].

## Abbreviations

API: Application Programming Interface; BWA: Burrows-Wheeler Aligner; CSV: comma-separated values; GATK: Genome Analysis Toolkit; Gb: gigabase pairs; GBS: genotyping-by-sequencing; IC4R: Information Commons for Rice; INDEL: insertion/deletion; NCBI: National Center for Biotechnology Information; NJ: neighbour-joining; RAM: random-access memory; SNP: single-nucleotide polymorphism; SRA: Sequence Read Archive; TSV: tab-separated value; VCF: variation call format; WEC: whole-exome-capture; WGS: whole-genome re-sequencing.

## Competing Interests

The authors declare that they have no competing interests.

## Funding

This work has been supported by the National Natural Science Foundation of China (grant No. 31701415), the National Key Research and Development Program of China (grant Nos. 2018YFD0100803 and 2016YFD0100801) and Chinese Universities Scientific Fund (grant No. 2019TC153).

## Authors’ Contributions

Method development: W.W., Z.W., and W.G.; implementation: W.W., Z.W., X.L., and W.G.; data preparation: Z.W.; design and testing: W.W., Z.W., Z.N., Z.H., M.X., H.P., Y.Y., Q.S., and W.G.; definition of research project: W.G.

## Supplementary Material

giaa060_GIGA-D-20-00003_Original_Submission

giaa060_GIGA-D-20-00003_Revision_1

giaa060_Response_to_Reviewer_Comments_Original_Submission

giaa060_Reviewer_1_Report_Original_Submission Armin Scheben -- 1/24/2020 Reviewed

giaa060_Reviewer_1_Report_Revision_1 Armin Scheben -- 4/12/2020 Reviewed

giaa060_Reviewer_2_Report_Original_Submission Awais Rasheed -- 3/5/2020 Reviewed

## References

[1] Zhou Z, Jiang Y, Wang Z, et al. Resequencing 302 wild and cultivated accessions identifies genes related to domestication and improvement in soybean. Nat Biotechnol. 2015;33:408–14. 25643055 10.1038/nbt.3096

[2] Chia J-M, Song C, Bradbury PJ, et al. Maize HapMap2 identifies extant variation from a genome in flux. Nat Genet. 2012;44:803–7. 22660545 10.1038/ng.2313

[3] Chapman JA, Mascher M, Buluç A, et al. A whole-genome shotgun approach for assembling and anchoring the hexaploid bread wheat genome. Genome Biol. 2015;16:26. 25637298 10.1186/s13059-015-0582-8 PMC4373400

[4] He F, Pasam R, Shi F, et al. Exome sequencing highlights the role of wild-relative introgression in shaping the adaptive landscape of the wheat genome. Nat Genet. 2019;51:896–904. 31043759 10.1038/s41588-019-0382-2

[5] Pont C, Leroy T, Seidel M, et al. Tracing the ancestry of modern bread wheats. Nat Genet. 2019;51:905–11. 31043760 10.1038/s41588-019-0393-z

[6] Cheng H, Liu J, Wen J, et al. Frequent intra- and inter-species introgression shapes the landscape of genetic variation in bread wheat. Genome Biol. 2019;20:136. 31300020 10.1186/s13059-019-1744-x PMC6624984

[7] Danecek P, Auton A, Abecasis G, et al. The variant call format and VCFtools. Bioinformatics. 2011;27:2156–8. 21653522 10.1093/bioinformatics/btr330 PMC3137218

[8] Hao L, Zhang HIC4R Project Consortium, et al., IC4R Project Consortium Information Commons for Rice (IC4R). Nucleic Acids Res. 2016;44:D1172–80. 26519466 10.1093/nar/gkv1141 PMC4702825

[9] Portwood JL, Woodhouse MR, Cannon EK, et al. MaizeGDB 2018: the maize multi-genome genetics and genomics database. Nucleic Acids Res. 2019;47:D1146–54. 30407532 10.1093/nar/gky1046 PMC6323944

[10] Mansueto L, Fuentes RR, Chebotarov D, et al. SNP-Seek II: A resource for allele mining and analysis of big genomic data in *Oryza sativa*. Curr Plant Biol. 2016;7-8:16–25.

[11] Ameur A, Bunikis I, Enroth S, et al. CanvasDB: a local database infrastructure for analysis of targeted- and whole genome re-sequencing projects. Database (Oxford). 2014;2014:bau098. 25281234 10.1093/database/bau098 PMC4184106

[12] Sempéré G, Pétel A, Rouard M, et al. Gigwa v2-Extended and improved genotype investigator. Gigascience. 2019;8, doi:10.1093/gigascience/giz051. PMC6511067 31077313

[13] Dereeper A, Homa F, Andres G, et al. SNiPlay3: a web-based application for exploration and large scale analyses of genomic variations. Nucleic Acids Res. 2015;43:W295–300. 26040700 10.1093/nar/gkv351 PMC4489301

[14] Docker-encapsulated version of SnpHub. https://github.com/esctrionsit/snphub4docker. Accessed 2nd June 2020.

[15] Li H, Handsaker B, Wysoker A, et al. The Sequence Alignment/Map format and SAMtools. Bioinformatics. 2009;25:2078–9. 19505943 10.1093/bioinformatics/btp352 PMC2723002

[16] Li H A statistical framework for SNP calling, mutation discovery, association mapping and population genetical parameter estimation from sequencing data. Bioinformatics. 2011;27:2987–93. 21903627 10.1093/bioinformatics/btr509 PMC3198575

[17] Shen W, Le S, Li Y, et al. SeqKit: A cross-platform and ultrafast toolkit for FASTA/Q file manipulation. PLoS One. 2016;11:e0163962. 27706213 10.1371/journal.pone.0163962 PMC5051824

[18] Li H Tabix: Fast retrieval of sequence features from generic TAB-delimited files. Bioinformatics. 2011;27:718–9. 21208982 10.1093/bioinformatics/btq671 PMC3042176

[19] Wickham H ggplot2. J R Stat Soc Ser A Stat Soc. 2016, doi:10.1007/978-3-319-24277-4.

[20] Kahle D, Wickham H ggmap: Spatial visualization with ggplot2. R J. 2013;5:144.

[21] dplyr. https://dplyr.tidyverse.org/. Accessed 2nd June 2020.

[22] rjson: JSON for R. https://cran.r-project.org/web/packages/rjson/index.html. Accessed 2nd June 2020.

[23] Paradis E pegas: an R package for population genetics with an integrated-modular approach. Bioinformatics. 2010;26:419–20. 20080509 10.1093/bioinformatics/btp696

[24] Knaus BJ, Grünwald NJ vcfr: a package to manipulate and visualize variant call format data in R. Mol Ecol Resour. 2017;17:44–53. 27401132 10.1111/1755-0998.12549

[25] Paradis E, Schliep K ape 5.0: an environment for modern phylogenetics and evolutionary analyses in R. Bioinformatics. 2019;35:526–8. 30016406 10.1093/bioinformatics/bty633

[26] An R Interface to the DataTables library. https://github.com/rstudio/DT. Accessed 2nd June 2020.

[27] Cingolani P, Platts A, Wang LL, et al. A program for annotating and predicting the effects of single nucleotide polymorphisms, SnpEff. Fly. 2012;6:80–92. 22728672 10.4161/fly.19695 PMC3679285

[28] Huerta-Sánchez E, Jin X, Asan BZ, et al. Altitude adaptation in Tibetans caused by introgression of Denisovan-like DNA. Nature. 2014;512:194–7. 25043035 10.1038/nature13408 PMC4134395

[29] Guo W, Zhu P, Pellegrini M, et al. CGmapTools improves the precision of heterozygous SNV calls and supports allele-specific methylation detection and visualization in bisulfite-sequencing data. Bioinformatics. 2018;34:381–7. 28968643 10.1093/bioinformatics/btx595 PMC6454434

[30] Buels R, Yao E, Diesh CM, et al. JBrowse: A dynamic web platform for genome visualization and analysis. Genome Biol. 2016;17:66. 27072794 10.1186/s13059-016-0924-1 PMC4830012

[31] Mayer KFX, Rogers J, Dole el J, et al. A chromosome-based draft sequence of the hexaploid bread wheat (*Triticum aestivum*) genome. Science. 2014;345:1251788. 25035500 10.1126/science.1251788

[32] Jordan KW, Wang S, Lun Y, et al. A haplotype map of allohexaploid wheat reveals distinct patterns of selection on homoeologous genomes. Genome Biol. 2015;16:48. 25886949 10.1186/s13059-015-0606-4 PMC4389885

[33] Wang H, Yin H, Jiao C, et al. Sympatric speciation of wild emmer wheat driven by ecology and chromosomal rearrangements. Proc Natl Acad Sci U S A. 2020;117:5955–63. 32123089 10.1073/pnas.1920415117 PMC7084162

[34] Avni R, Nave M, Barad O, et al. Wild emmer genome architecture and diversity elucidate wheat evolution and domestication. Science. 2017;357:93–7. 28684525 10.1126/science.aan0032

[35] Singh N, Wu S, Tiwari V, et al. Genomic analysis confirms population structure and identifies inter-lineage hybrids in *Aegilops tauschii*. Front Plant Sci. 2019;10:9. 30740115 10.3389/fpls.2019.00009 PMC6357674

[36] Bolger AM, Lohse M, Usadel B Trimmomatic: A flexible trimmer for Illumina sequence data. Bioinformatics. 2014;30:2114–20. 24695404 10.1093/bioinformatics/btu170 PMC4103590

[37] Li H Aligning sequence reads, clone sequences and assembly contigs with BWA-MEM. arXiv. 2013:1303.3997.

[38] McKenna A, Hanna M, Banks E, et al. The Genome Analysis Toolkit: A MapReduce framework for analyzing next-generation DNA sequencing data. Genome Res. 2010;20:1297–303. 20644199 10.1101/gr.107524.110 PMC2928508

[39] Wang W, Wang Z, Li X, et al. Supporting data for “SnpHub: an easy-to-set-up web server framework for exploring large-scale genomic variation data in the post-genomic era with applications in wheat.”. GigaScience Database. 2020, 10.5524/100745. PMC7274028 32501478

