# Supplementary material for: SnpHub: an easy-to-set-up web server framework for exploring large-scale genomic variation data in the post-genomic era with applications in wheat
Source: Gigascience. 2020 Jun 5;9(6):giaa060. doi: 10.1093/gigascience/giaa060 (PMC7274028; doi:10.1093/gigascience/giaa060)
Supplement: giaa060_GIGA-D-20-00003_Revision_1 [file giaa060_giga-d-20-00003_revision_1.pdf]

GigaScience

SnpHub: an easy-to-set-up web server framework for exploring large-scale genomic variation data in the post-genomic era with applications in wheat

--Manuscript Draft--

**Manuscript Number:**	GIGA-D-20-00003R1
**Full Title:**	SnpHub: an easy-to-set-up web server framework for exploring large-scale genomic variation data in the post-genomic era with applications in wheat
**Article Type:**	Technical Note
**Funding Information:**	National Natural Science Foundation of China (31701415) — Dr. Weilong Guo<br>National Key Research and Development Program of China (2018YFD0100803) — Dr. Weilong Guo<br>National Key Research and Development Program of China (2016YFD0100801) — Dr. Zhongfu Ni
**Abstract:**	Background: The cost of high-throughput sequencing is rapidly decreasing, allowing researchers to investigate genomic variations across hundreds or even thousands of samples in the post-genomic era. The management and exploration of these large-scale genomic variation data require programming skills. The public genotype querying databases of many species are usually centralized and implemented independently, making them difficult to update with new data over time. Currently, there is a lack of a widely used framework for setting up user-friendly web servers for exploring new genomic variation data in diverse species.<br>Results: Here, we present SnpHub, a Shiny/R-based server framework for retrieving, analysing and visualizing the large-scale genomic variation data that be easily set up on any Linux server. After a pre-building process based on the provided VCF files and genome annotation files, the local server allows users to interactively access SNPs/INDELs and annotation information by locus or gene and for user-defined sample sets through a webpage. Users can freely analyse and visualize genomic variations in heatmaps, phylogenetic trees, haplotype networks, or geographical maps. Sample-specific sequences can be accessed as replaced by SNPs/INDELs.<br>Conclusions: SnpHub can be applied to any species, and we build up a SnpHub portal website for wheat and its progenitors based on published data in present studies. SnpHub and its tutorial are available as http://guoweilong.github.io/SnpHub/.
**Corresponding Author:**	Weilong Guo, PhD<br><br>CHINA
**Corresponding Author Secondary Information:**	
**Corresponding Author's Institution:**	
**Corresponding Author's Secondary Institution:**	
**First Author:**	Wenxi Wang
**First Author Secondary Information:**	
**Order of Authors:**	Wenxi Wang
	Zihao Wang
	Xintong Li
	Zhongfu Ni
	Zhaorong Hu
	Mingming Xin
	Huiru Peng

	Yingyin Yao
	Qixin Sun
	Weilong Guo, PhD
**Order of Authors Secondary Information:**	
**Response to Reviewers:**	Dear Editor,<br><br>We are very thankful to you and all the reviewers for your constructive comments to help improve our manuscript "SnpHub: an easy-to-set-up web server framework for exploring large-scale genomic variation data in the post-genomic era with applications in wheat" (GIGA-D-20-00003). We have considered all comments and suggestions and carefully revised the manuscript.<br><br>Generally, our modifications include the following main aspects:<br>1. A comparison between SnpHub and other three similar applications (Gigwa v2, CanvasDB and JBrowse) have been added, to help user decide on when to use SnpHub based on their actual needs. And new table for functional comparison (Table 1) were added in the revised manuscirpt.<br>2. As Wang et al. recently published new dataset on wild emmer wheat, we currently have included this dataset in Wheat-SnpHub-Portal database. The details in the Table2 (original Table 1) were also edited to be consistent with http://wheat.cau.edu.cn/Wheat_SnpHub_Portal/.<br>3. A new paragraph is added for explaining how data behind Wheat-SnpHub-Portal were prepared to get the VCF files.<br>4. We have updated SnpHub homepage and tutorial webpage, by correcting the links, fixed typos, rewrite to improve the grammars, and also have added animation illustrations for each function in forms of GIF figures.<br>5. We have included in SnpHub with support for integrating microarray data in hapmap format.<br>6. All the R packages and tools utilized in SnpHub have been cited or listed with URLs in the revised manuscript.<br>7. We have registered SnpHub in the bio.tools and SciCrunch.org databases, and have provided RRID and biotoolsID identifiers in the section of "Availability of supporting source code and requirements".<br><br>The point-by-point response to comments and questions from the reviewers point-by-point are also attached.<br><br>Thank you again for all of your assistance.<br><br>Sincerely,<br>Weilong Guo<br><br><br>************************<br>REVIEWS<br>************************<br>Reviewer #1:<br><br>The authors present their software SnpHub for data exploration of VCF files. The software is a useful contribution to the crop genomics community and has brought together a number of existing tools to provide a range of features in a Shiny/R framework. The authors provide a web page with installation instructions and guides on usage. I have two main comments for the authors that I think should be addressed.<br><br>Reply: Thanks for the reviewer's positive comments. We have substantially revised the manuscript upon these valuable suggestions.<br><br>1) Although the authors point out that similar tools such as Gigwa v2 and CanvasDB exist, the comparison seems cursory. The authors report that the main benefit of SnpHub is more efficient management of variant data, but this is not further supported. For example, the table (Table 1) showing the disk usage of the wheat data sets does not compare the potential disk usage if using other tools to build a queryable database. The manuscript may benefit from some further comparison that would allow readers to decide on when to use SnpHub, rather than other tools, based on their specific needs.

Powered by Editorial Manager® and ProduXion Manager® from Aries Systems Corporation

Reply: We thank the reviewer for this beneficial suggestion. We agree that providing such a comparison will be useful for readers. We have added a new paragraph and a new table (Table 1 in revised manuscript) for comparing SnpHub with Gigwa v2, CanvasDB and JBrowse.

The corresponding revised paragraph and new table run as following:

“Advantages of SnpHub in managing variation data
SnpHub is designed as a database framework specialized for retrieving and light-weighted analysis of large genomic variation data. To provide instant responses for queries and interactive analysis, SnpHub focuses on the supports for haplotype analysis or genomic variation analysis for specific region or gene, rather than genome-wide scale analysis such as GWAS analysis. For a clear view on the advantages of SnpHub, a comprehensive comparison on their supported features is presented (Table 1) with three other popular frameworks. Both Gigwa v2 [12] and CanvasDB [11] are specialized framework for investigating genotype data, and are implemented with SQL-based database engines. The SQL-based servers generally require reload genotype information into specialized database tables, and meanwhile lost the resourceful meta-information in VCF files for describing variations. SnpHub is actually based on BCF format, which is lossless binary converted format of VCF and wildly used by bioinformaticians, and thus will save disk storage in practice. JBrowse [26] is general-purpose genome browser framework, and provide flexible visualization and querying functions, while with shortcoming in support of re-analyses. In contrast, SnpHub is designed with R/Shiny framework, providing a variety of both visualization and re-analysis functions. Moreover, as R packages and R/Shiny framework are wildly accepted by the bioinformatician communities, it would be easier for SnpHub to incorporate powerful analysis function and be extended. In general, SnpHub allowed users an alternative choice for interactively exploring the huge genomic diversity data and are more strengthen in performing light-weight re-analyses, including group-wise comparison, haplotype-related analysis, phylogenetic analysis, passport visualization, retrieving consensus sequences and generating processable tables and figures.”

2) I could not access the SnpHub Wheat Portal using the provided link (http://wheat.cau.edu.cn/Wheat_SnpHub_Portal/), making it difficult to evaluate this aspect of the paper. As the wheat portal is also a resource presented by the paper, this should be available. I had the same issue after attempting to access the link on different days and using different browsers (Chrome and Firefox), though I cannot rule out that the issue was on my side.

Reply: We thank the reviewers for letting us be aware of the inaccessible of the Wheat-SnpHub-Portal. We have checked about it and found that there has been a two weeks reconstruction of campus network, which making it failed to access our new website outside the campus, while it works well within the campus. Currently, the problem is solved, and should be able to accessed globally.
Moreover, to avoid such case in the future, we have added monitoring server from proxy to keep us aware with accessible status from outside. Email feedbacks for accessing the server are welcomed in the future, and we’ll try the best to keep it accessible.

Minor comments
3) The authors may want to cite all of the bioinformatics software which their tool relies on. On page 6 the authors write "Several widely used bioinformatics software programs must be pre-installed, such as SAMtools [14], bcftools [15], seqkit [16] and Tabix [17], along with several R packages". These R packages should be cited, particularly if they have been published in scientific journals as, for example, vcfR has (Knaus, Brian J., and Niklaus J. Grunwald. 2017. VCFR: a package to manipulate and visualize variant call format data in R. Molecular Ecology Resources 17(1):44-53).

Reply: We thank the reviewer for the valuable suggestions. We have gone through the manuscript and tried the best to add citations for published softwares, those SnpHub relies on, including ggplot2, ggmap, pegas, vcfR, ape, etc. For the packages or softwares has not been published in scientific journals, we also have added the URLs for accessing the source code.

4) Pg 14: The authors state "We downloaded all the above published datasets (Table 1), and then generated VCF files from raw sequencing data or utilized the published VCF files directly." Please add detail of how the VCFs were generated. If the data is meant to be used as a resource, it must be clear to users how it was generated.

Powered by Editorial Manager® and ProduXion Manager® from Aries Systems Corporation

Reply: We thank the reviewer for this concern. We have added a corresponding paragraph for describing the generation process of VCF files with detailed parameters. And a sentence is added for connecting two paragraphs.
The added descriptions of VCF file generation run as follows:

"Using all the above published datasets (Table 2), we constructed up the "Wheat-SnpHub-Portal" website. The VCF files from He et al. [4] and Pont et al. [5] were downloaded from the links provided in their original papers. For datasets from Cheng et al. [6] and Wang et al. [29], the genotyping data in VCF formats were shared by the authors. For dataset from Singh et al. [31], raw sequence reads were downloaded from NCBI SRA under accession SRP141206 and VCF files were regenerated using scripts provided in the paper. As for dataset of Jordan et al. [28] and Avni et al. [30], we downloaded raw sequence data from NCBI SRA under SRP167848 and SRP032974, respectively. Raw reads were then trimmed using Trimmomatic [32] and aligned to reference genomes using BWA-MEM [33]. SNPs and INDELs were identified with HaplotypeCaller module of GATK [34] and were further filtered by VariantFiltration function with the parameter "QD<2.0 || FS>60.0 || MQRankSum<−12.5 || ReadPosRankSum<− 8.0 || SOR>3.0 || MQ< 40.0 || DP >30 || DP < 3." and "QD< 2.0 || FS>200.0 || ReadPosRankSum<−20.0 || DP>30 || DP< 3", respectively. Generally, with the provided configuration data and variation files, the pre-processing step can be quickly finished, taking from ~8 minutes [5] to ~4 hours [6]."

5) There are some minor errors in the web pages for snphub, so it may be worthwhile going over some of these. Two of the errors I found were as follows. I think making sure that the installation and set up go as smoothly as possible, particularly for the biologists without a programming background that SnpHub is aimed at, will be an important aspect of helping this tool get taken up by the community.
On the github page (https://github.com/esctrionsit/snphub) the authors state "Edit the setup_config.R file, make sure all the paths are correct." However the file "setup_config.R" does not exist in the github repo, instead I think the file is called "setup.R".
The github link on the top of the quick start description (https://esctrionsit.github.io/snphub_tutorial/content/Setup/quickstart.html) is broken for me.

Reply: We thank the reviewer for figuring out these inconsistence and mistakes in the document webpage, which is created due to the recent updates in documents and websites. We have corrected the links in homepages, double checked the accessibility of all links in our websites, and also have tried best to corrected the tyros and grammar mistakes in the documents.

For the issue with "setup_config.R", the configuration file of SnpHub has been renamed from "setup_config.R" to "setup.conf", to make it more explicit to users. The SnpHub homepage (https://guoweilong.github.io/SnpHub/) and tutorial webpage (https://esctrionsit.github.io/snphub_tutorial/) have both been updated.
The documents for "general setup" can be found at https://esctrionsit.github.io/snphub_tutorial/content/Setup/quick_deploy.html.
The documents for "quick setup with Docker" can be found at https://esctrionsit.github.io/snphub_tutorial/content/Docker/overview.html .

****
Reviewer #2:

The publication and the resource developed for has merits and provided handy tools for analysis of SNP data.
1) A major shortcoming is the analysis of the SNP array data. Can they include an interface to analyse SNP array data, where the data is usually available in .hapmap format.

Reply: We thank the reviewer for this useful suggestion. We have added the support for importing the SNP array data which are usually available in .hapmap format.
In the latest version of SnpHub, We have added an utility to convert hapmap format to VCF. Such format conversion can be done with following command.

```
python snphub/data_transfer/hapmap2vcf.py -i [input hapmap path] -o [output vcf path]
```

Then the new generated VCF files can be feed with the whole SnpHub pipelines. And we have also updated this new feature on the tutorial webpage

Powered by Editorial Manager® and ProduXion Manager® from Aries Systems Corporation

(https://esctrionsit.github.io/snphub_tutorial/content/QA/QA.html#inputoutput-formats).

2) I will suggest to add a video tutorial for using snpHub. Please see a recent rice galaxy paper published in Gigascience. It would greatly help breeding community, which sometime don't have expertise for such analyses.

Reply: We thank the reviewer for this suggestion. We agree with the reviewer on improving the tutorial by adding illustration videos. While considering the compatibility with our documenting system, we currently decide to add animation figures (in GIF format) to illustrate the usage of document website. We have recorded the basic usage of each function as GIFs, illustrating how to click and fill the parameters, and what would be shown in output as the examples.
Here is the link for one example:
https://esctrionsit.github.io/snphub_tutorial/content/Basic_Usage/vartable.html#demonstration

3) No bioinformatics pipeline is embedded in the resource, this could be easily incorporated to add a blast tool or annotation of wheat genome sequence.

Reply: Actually, we have included many bioinformatics analysis functions in SnpHub, including heatmap, haplotype-network analysis and construction of phylogenetic trees. For the annotation of variations, the VCF file will be annotated by SnpEff using provided GFF3 file in our pre-processing step, thus annotation information can be displayed in "VarTable", "Heatmap" and "SnpFreq" functions.
As we have described in the manuscript, Snphub is designed as a database framework specialized for management the querying for genomic variation data, with advantages in managing, supporting querying and reanalysis for SNP and indels.
Some bioinformatics functions, such as blast and query for gene functions, are supposed to be useful, and have already been implemented or integrated in general-purpose databases. These applications can be widely found, and have few connections with the design purpose of SnpHub. We would not plan to add such function in the framework of SnpHub. However, we believe it would be useful for building the comprehensive database, such as the I (http://202.194.139.32/).
Moreover, we added a clarification for describing the scope of Snphub in the revised manuscript. It runs as following:
"SnpHub is designed as a database framework specialized for retrieving and light-weighted analysis of large genomic variation data."

4) Since it is more focused to be a visualization tool, I would suggest if the variants could be visualized in wheat JBrowse.

Reply: We appreciate the reviewer providing this suggestion.
Actually, SnpHub is an independent database framework with JBrowse. And many designed functions of SnpHub are complementary with the functions of JBrowse. The implemented strategies of SnpHub and JBrowse are different. For example, JBrowse forcus on the visualization of variations through tracked view, and SnpHub forcus on data management, data retrieving and data re-analyzation together with visualization of some results.
In the revised manuscript, we have added the following descriptions for comparing SnpHub and JBrowse. And we also included JBrowse for feature comparison, as suggested the reviewer #1.

"JBrowse [26] is general-purpose genome browser framework, and provide flexible visualization and querying functions, while with shortcoming in support of re-analyses. In contrast, SnpHub is designed with R/Shiny framework, providing a variety of both visualization and re-analysis functions. Moreover, as R packages and R/Shiny framework are wildly accepted by the bioinformatician communities, it would be easier for SnpHub to incorporate powerful analysis function and be extended."

Additional Information:	
Question	Response
Are you submitting this manuscript to a special series or article collection?	No

Powered by Editorial Manager® and ProduXion Manager® from Aries Systems Corporation

**Experimental design and statistics**<br><br>Full details of the experimental design and statistical methods used should be given in the Methods section, as detailed in our Minimum Standards Reporting Checklist. Information essential to interpreting the data presented should be made available in the figure legends.<br><br>Have you included all the information requested in your manuscript?	Yes
**Resources**<br><br>A description of all resources used, including antibodies, cell lines, animals and software tools, with enough information to allow them to be uniquely identified, should be included in the Methods section. Authors are strongly encouraged to cite Research Resource Identifiers (RRIDs) for antibodies, model organisms and tools, where possible.<br><br>Have you included the information requested as detailed in our Minimum Standards Reporting Checklist?	Yes
**Availability of data and materials**<br><br>All datasets and code on which the conclusions of the paper rely must be either included in your submission or deposited in publicly available repositories (where available and ethically appropriate), referencing such data using a unique identifier in the references and in the “Availability of Data and Materials” section of your manuscript.<br><br>Have you have met the above requirement as detailed in our Minimum Standards Reporting Checklist?	Yes

Powered by Editorial Manager® and ProduXion Manager® from Aries Systems Corporation

Manuscript

Click here to access/download;Manuscript;ms20200404_revised_for_produc

SnpHub: an easy-to-set-up web server framework for exploring large-scale genomic variation data in the post-genomic era with applications in wheat

Wenxi Wang[1,†], Zihao Wang[1,†], Xintong Li[1], Zhongfu Ni[1], Zhaorong Hu[1], Mingming Xin[1], Huiru Peng[1], Yingyin Yao[1], Qixin Sun[1] and Weilong Guo[1,*]

[1]Key Laboratory of Crop Heterosis and Utilization, State Key Laboratory for Agrobiotechnology, Beijing Key Laboratory of Crop Genetic Improvement, China Agricultural University, Beijing 100193, China.

* Correspondence address. Weilong Guo, Beijing, China. E-mail: guoweilong@cau.edu.cn

†The authors contributed equally to this work.

Abstract

Background: The cost of high-throughput sequencing is rapidly decreasing, allowing researchers to investigate genomic variations across hundreds or even thousands of samples in the post-genomic era. The management and exploration of these large-scale genomic variation data require programming skills. The public genotype querying databases of many species are usually centralized and implemented independently, making them difficult to update with new data over time. Currently, there is a lack of a widely used framework for setting up user-friendly web servers for exploring new genomic variation data in diverse species.

Results: Here, we present SnpHub, a Shiny/R-based server framework for retrieving, analysing and visualizing the large-scale genomic variation data that be easily set up on any Linux server. After a pre-building process based

on the provided VCF files and genome annotation files, the local server allows users to interactively access SNPs/INDELs and annotation information by locus or gene and for user-defined sample sets through a webpage. Users can freely analyse and visualize genomic variations in heatmaps, phylogenetic trees, haplotype networks, or geographical maps. Sample-specific sequences can be accessed as replaced by SNPs/INDELs.

Conclusions: SnpHub can be applied to any species, and we build up a SnpHub portal website for wheat and its progenitors based on published data in present studies. SnpHub and its tutorial are available as http://guoweilong.github.io/SnpHub/.

Keywords: SNP, database, server-framework, R/Shiny, wheat

Introduction

Competition in the field of high-throughput sequencing greatly contributes to the reduction of sequencing costs. Currently, one thousand dollars is the cost of sequencing approximately 5 human genomes, 1 hexaploid wheat genome, 6 maize genomes or 50 rice genomes at an average depth of 10×. Whole-genome sequencing is commonly used for species with mid-sized genome such as soybean [1] and maize [2]. Genotyping-by-sequencing (GBS) or whole-genome exon-capture sequencing (WEC) technologies are also frequently used for large-genome species, such as wheat [3]. Currently, many wheat genome studies profile genomic variations on a scale of hundreds or thousands of accessions through WEC [4,5] or whole-genome re-sequencing (WGS) [6].

Plant sciences have experienced a dramatic increase in the available genomic variation data due to the assessment of diverse species and plentiful germplasm resources. Beyond investigating the genetic diversity among individuals, large panels of high-quality genomic variation data have provided valuable resources and great opportunities for identifying trait-related genes, designing markers, constructing gene trees, exploring the evolutionary history and assisting design of molecular breeding. Low-depth re-sequencing data from recombinant inbred line (RIL) populations can assist in the identification of quantitative trait loci (QTLs) for traits of interest. Profiling the genomic variation of TILLING populations in crop studies can benefit the exploration of candidate variations that are rare in nature. The re-use of genomic variation data plays an important role in driving current plant science research.

As a routing pipeline, the raw reads obtained in whole-genome sequencing are first aligned to reference genomes. Then, SNPs and small insertions/deletions (INDELs) are called and stored in the standard variation call format (VCF) files [7]. Although there are great numbers of command-line tools for bioinformaticians to manage and process VCF files, these files are usually quite large. The efficient management of the massive accumulated genomic sequencing data and exploration of these large-scale genomic variation data require computational skills, exceeding the abilities of most biologists.

Some public databases are available for querying sample-specific genomic variations, such as the IC4R database for rice studies [8] and MaizeGDB for maize studies [9]. These public databases are usually based on re-sequencing data, that are generated and maintained by large

international consortia. The web servers are implemented independently, providing different functions in exploring the genomic variations. With the increasing number of researchers from generating new data worldwide, it has become impossible to maintain a centralized database that is both up-to-date and comprehensive. There is great demand for implementing a universal platform for building distributed or private web servers for genomic data querying.

Several attempts have been made to implement web-application frameworks. SNP-Seek II creates HDF files for storing genotypic data and utilizes Java Spring and ZK frameworks for implementing the web-application architecture [10]. SNP-Seek II mainly supports data retrieval but is mainly designed for rice studies, and maintaining the complex computing structure requires professional technicians. CanvasDB is designed as a local database infrastructure for managing and retrieving the variation data using the MySQL database and supports filtering functionality and variation detection using R functions [11]. Gigwa v2 also imports VCF files in the NoSQL database, providing both analysis and visualization functions [12]. However, because relational databases are designed for table-structured data, systems such as MySQL are not the optimal method for managing complex genomic variation information, and uncompressed genomic variation data usually require a large amount of memory. SNiPlay3 is based on the Galaxy framework and provides a panel analysis that mainly focuses on whole-genome studies [13]. However, with the rapid accumulation of self-organized genomic variation data, there are still gaps in meeting the great requirements for a uniform, user-friendly, powerful web server framework to with fast and efficient access to massive

genomic variation data both locally and in a centralized location, to allow biologists to investigate genomic variations without the need for programming skills.

Here, we developed SnpHub as a uniform web server framework that can be easily set up locally and can be applied by researchers for conveniently management of the massive processed VCF files and used to interactively explore the genomic diversity and perform rapidly analyses in their own labs. SnpHub is designed for rapidly accessing SNP/INDEL data from specific regions and specific sample groups, rather than performing whole-genome analysis. This framework is designed to be species independent, to support scalable variation data and to provide resourceful and extendable functions for re-using and re-analysing genomic variation data.

Methods

The general SnpHub framework

The SnpHub framework is designed to be installed in the Linux system, utilizing the Shiny/R framework and integrating several widely used bioinformatics command-line software packages and R packages for analysing and processing genotyping data. SnpHub can be efficiently hosted on a modest computing server, with a local computer installed with R-studio. Rather than performing a whole-genome general analysis, SnpHub provides an efficient way to quickly access data in a local region, filter sites and samples, and generate a genotype table as the intermedia data. To enhance the performance of re-using SNP/INDEL data for in-depth exploration, the

intermedia genotype table is stored in random-access memory (RAM) and then used for subsequent analyses and visualizations (Figure 1).

The interactive user interface is implemented using the R/Shiny framework, with powerful, convenient functions for post-processing and visualizing the genotyping data. Considering that a large proportion of open-source bioinformatics analysis tools are implemented using R, SnpHub utilizes the R/Shiny framework to make it compatible and extendable. For simplify installation, a wrapped-version with deploying the SnpHub Docker container is provided at https://github.com/esctrionsit/snphub4docker.

The setup of SnpHub

Prepare step

The SnpHub server-framework is designed to be lightweight and to rapidly access query information from the massive data stored on hard disks while requiring very little RAM. A general Linux workstation (for example, 4G RAM and 2.3 GHz dual process) installed with Shiny/R is enough to set up an instance of SnpHub. Several widely used bioinformatics software programs must be pre-installed, such as SAMtools [14], bcftools [15], seqkit [16] and Tabix [17], along with several R packages, such as ggplot2 [18], ggmap [19], dplyr (https://dplyr.tidyverse.org/), rjson(https://cran.r-project.org/web/packages/rjson/index.html), pegas [20], vcfR [21], ape [22], DT(https://github.com/rstudio/DT).

To build a local instance, the VCF files, reference genome sequence file (FASTA format), gene annotation file (gff3 format) and metadata files defining sample information (tab-separated value, TSV format) are needed. Providing meta-information such as sample information and pre-defined sample groups

will enhance performance. A configuration template is provided for presenting meta-information such as the species name, sample description, reference genome, alignment method, and source of the accession.

Pre-processing step

A shell wrapper program is provided for the pre-building process. Once an instance server is built, users can access the data through a web browser interactively. Once the configuration information is provided, the local SnpHub instance can be easily built by running the shell-wrapper in one command-line. SnpHub will check the system environment for essential software and the formats of provided files. Then, the gene-based annotation of SNPs/INDELs will be performed by SnpEff [23]. All the meta-information is stored as tables on the hard disks, which is achieved by Tabix [17] for fast retrieval of the content.

Key features for improving the performance of SnpHub

Rapid retrieval of genotype matrix by randomly accessing the disk

Considering that a relational database such as the mySQL framework is suitable for tables and requires a large amount of memory, SnpHub instead adopts the bioinformatician-friendly BCF format for storing the massive genomic variation data. BCF is a binary file format corresponding to VCF [15] with improved performance for supporting the fast querying of a subset of data by randomly accessing the hard disk, taking advantage of the BGZF compression format. In the pre-processing step, all the VCF files will be converted to BCF files. Another benefit is that bioinformaticians can directly perform analysis on these BCF files without storing another copy or format for the same dataset. To improve performance, SnpHub only retrieves a small

piece of data for the selected region and selected samples from the disk instantly and stores the intermediate SNP/INDEL table in RAM to be efficiently processed by the downstream analysis functions.

The triple-name strategy balances convenience and efficiency

To balance the requirements of convenience in management by server managers, ease of querying and readability of the analysis result, SnpHub utilizes a triple-name/ID for a sample, which includes (a) the *vcfID*, (b) the *Accession name* and (c) the *Display name*. The *vcfID* is a string name that is the same as that provided in the VCF files, avoiding the modification of the original VCF files. The *Accession name* is usually a short name, such as "*S01,S02,S03*", so that it can be easily typed in the input box for querying a list of samples. The *Display name* is designed as a readable name to be displayed in the results and figures so that researchers can conveniently interpret the result. Arbitrary sample information such as sample passport or sample notes can be provided in additional columns. Once the SnpHub instance is set up, a sample information webpage with a search engine is provided for navigating the names of the available samples and corresponding meta-information.

Analysis with defined sample groups

A new feature of SnpHub is that it allows the querying of samples by groups, either using a pre-defined groupID or defining new groups. When setting up the server instance, the database manager can define the system-wide groupIDs by configuring the TSV file. Then, the users can conveniently use groupIDs for querying a list of genes such as #*groupID*. Also, the user can define a custom groupID for a list of samples with the syntax such as

NewGroupA{Sample1,Sample2,Sample3}. With the defined groups, it will be convenient to refer a list of samples using one GroupID instead of the full list of sample names. By default, SnpHub reserves the group ID “*#ALL*” for querying all the samples in the cohort.

Exporting the tables and figures

SnpHub allows users to export tables in CSV format. Beyond interactive visualization of data by the many analysis tools, all the figures can be exported in both PNG and PDF formats. The exported PDF figure represents the vector graphic, as users can conveniently post-edit the figures using tools such as *Illustrator*. A panel of parameters is provided for formulating the height and width of exported figures to produce a satisfactory layout. To be reproducible and traceable, the time-stamp and main parameters are appended to the exported figures.

Results

Main functions provided by SnpHub

SnpHub supports the navigation of massive genomic variation data by users by specifying a list of samples and specific genomic regions and performing lightweight analyses and visualizations through the Shiny/R framework. Uniform, flexible interfaces for manipulating the query parameters are provided. As many open-source bioinformatics tools are implemented as command lines or R packages, the Shiny/R framework could be extended for integrating new tools for processing genomic variation tables. SnpHub provides user-friendly functions for navigating genomic variation data by implementing each of the functions on an independent tab page (Figure 2).

Raw variation data and genomic sequence retrieval functions are provided in VarTable and SeqMaker. Versatile analysis and visualization functions are provided, including Heatmap, HapNet, PhyloTree, SnpFreq and HapMap. In all of these functions, SnpHub directly queries a gene ID as the corresponding genomic region directly based on the provided GFF file.

VarTable, for exporting region-specific variation tables

In the ***VarTable*** function, users can query gene-/region-specific SNP/INDEL tables for a list of samples. To be consistent with the VCF format, the exported genotypes are denoted as “0/0”, “1/1”, “0/1” or “./.”, representing the “same genotype with reference genome”, “homozygous variant genotype”, “heterozygous genotype”, or “missing data”, respectively. Tables can be downloaded as raw data or as specific genotypes. This function supports a panel of parameters for filtering sites, such as the minimum allele frequency (MAF) and the maximum missing data frequency. To specify a region, users can either provide a specific region such as “*chr:startPos-endPos*” or use a geneID together with a parameter for the length of the flanking region. To support different purposes for discovering interesting variations, SnpHub extends the sample-based filtering interface to three categories : (a) samples must exhibit genotype variations compared with the reference genome; (b) samples must be consistent with the reference genome in their genotype; and (c) samples shall be exported in parallel without filtering on the basis of genotypes. Beyond the genotype, the meta-data that are stored in VCF format, such as the read depth (DP), genotype quality (GQ) and variant annotations generated by SnpEff (ANN), can also be exported when the optional parameter boxes are checked.

Heatmap, for visualizing genotypes in a matrix

The ***Heatmap*** function is an intuitive way to visualize tabular genotype information as a heatmap graph. The samples to be visualized can be provided in one group or a list of groups. By default, genomic positions are displayed along rows, and samples are displayed in columns. The parameters of the two dimensions can be exchanged. Different colours are used to represent homozygous mutations, heterozygous mutations, reference genotypes, and missing data. To be more intuitively visualize possible haplotypes, samples are clustered within each group according to their genotype similarity. This function can be useful for exploring group-specific haplotypes or genotype patterns.

HapNet, for constructing a haplotype network

The ***HapNet*** function provides an interface for constructing a haplotype network, which is widely used for characterizing the relationships among population based on sequences. The R package pegas [20] is used for generating the median-joining haplotype network plots. In the HapNet plot, each node represents a haplotype, whose radius is proportional to the number of samples harbouring this haplotype. The distance matrix is calculated among haplotypes based on their sequence distances. Finally, a minimum spanning tree (MST) is constructed. If multiple groups are provided, the nodes will be extended to a pie chart showing the proportion of each group. Similar haplotypes are joined by edges, with the distance shown on the edges. The haplotype network is usually used for exploring the evolutionary paths of different haplotypes among groups of samples [24].

PhyloTree, for visualizing sample distance in a local region

The ***PhyloTree*** function supports the exploration of the gene-based genetic distances and evolutionary history based on high-density SNP data. The distance matrix is calculated based on the genetic distances of specified genomic region. Then, two distance-based clustering methods, neighbour-joining (NJ) tree analysis and multidimensional scaling (MDS, also known as PCoA), are supported. NJ-tree analysis cab rapidly evaluate a large amount of data and is suitable for exploring the genetic relationships among samples for a specific region with a low time cost. Versatile layouts for visualizing the NJ-trees are available, including phylogram, cladogram, unrooted, fan, and radial layouts. Samples in different groups are shown in different colours. The MDS analysis supports the visualization of the distances of samples in two-dimensions through non-linear dimensionality reduction. This function provides users with multiple ways to visualize the sample distances for a local region.

SnpFreq, for visualizing the SNP annotation in lollipop format

The ***SnpFreq*** function allows users to visualize the SNPs/INDELs and functional annotations along with the transcript-tracks. The previously proposed Lollipop graph [25] is adopted to visualize the positions and frequencies of genomic variations to distinguish the low-frequency variants and un-detected variations. Variants causing amino acid changes are annotated in different colours, including missense variation, synonymous variant, frameshift variant, stop code gained/lost and splice region variants. Transcripts in the same region are displayed as different tracks at the bottom, indicating the exons, introns, CDSs and transcription directions. Samples in different groups are summarized independently and visualized in different

tracks, which can be useful for exploring the different frequencies of SNPs between groups.

HapMap, for visualizing the genotypes geographically

The ***HapMap*** function provides a way to project the allele distribution of a single site geographically on a map, utilizing the provided resource-gathering locations. A specific genomic site is required for the querying input boxes, such as "*chr:pos*". If a genomic region is provided, the first variant site in this region will be used for the analysis. To increase user friendliness, this function allows users to adjust the ranges of both longitudes and latitudes. A parameter is provided for the user to select the proper distance for merging geographically closely distributed accessions in one circle. This function could help to shed light on the spreading paths or histories of certain genomic variations/haplotypes.

SeqMaker, for creating consensus sequence for an individual

The ***SeqMaker*** function can help to create a consensus sequence by substituting variants based on the reference genome, and the result can be downloaded directly as FASTA file. In principle, this function retrieves a sample-specific sequence by replacing the detected genomic variations, which could be useful for sequence comparisons or primer design. However, it should be noted that the consensus sequences may not reflect the real sequences, considering the missing data as a result of sequencing coverages. Additionally, large structural variants are usually difficult to detect by re-sequencing. By default, "*#RAW*" is preserved for retrieving the raw sequence in the reference genome.

Advantages of SnpHub in managing variation data

SnpHub is designed as a database framework specialized for retrieving and light-weighted analysis of large genomic variation data. To provide instant responses for queries and interactive analysis, SnpHub focuses on the supports for haplotype analysis or genomic variation analysis for specific region or gene, rather than genome-wide scale analysis such as GWAS analysis. For a clear view on the advantages of SnpHub, a comprehensive comparison on their supported features is presented (Table 1) with three other popular frameworks. Both Gigwa v2 [12] and CanvasDB [11] are specialized framework for investigating genotype data, and are implemented with SQL-based database engines. The SQL-based servers generally require reload genotype information into specialized database tables, and meanwhile lost the resourceful meta-information in VCF files for describing variations. SnpHub is actually based on BCF format, which is lossless binary converted format of VCF and wildly used by bioinformaticians, and thus will save disk storage in practice. JBrowse [26] is general-purpose genome browser framework, and provide flexible visualization and querying functions, while with shortcoming in support of re-analyses. In contrast, SnpHub is designed with R/Shiny framework, providing a variety of both visualization and re-analysis functions. Moreover, as R packages and R/Shiny framework are wildly accepted by the bioinformatician communities, it would be easier for SnpHub to incorporate powerful analysis function and be extended. In general, SnpHub allowed users an alternative choice for interactively exploring the huge genomic diversity data and are more strengthen in performing light-weight re-analyses, including group-wise comparison, haplotype-related analysis, phylogenetic

analysis, passport visualization, retrieving consensus sequences and generating processable tables and figures.

Construction of the Wheat-SnpHub-Portal by SnpHub

Bread wheat is one of the most important staple crops and exhibits a large, repetitive genome whose genome size is estimated to be ~16 Gbp. As a hexaploid plant, bread wheat has a complex polyploidization history [27]. Following the release of high-quality reference genomes of wheat and its progenitors, a number of population genomics studies were released together with raw sequencing data or genomic variation data. Jordan et al. sequenced 62 lines of bread wheat (AABBDD) using WEC and GBS methods [28]. Two large WEC-based wheat population genomic studies sequenced 1026 lines [4] and 487 lines [5]. Recently, Cheng et al. performed a high-resolution resequencing study of 93 hexaploidy wheat lines [6]. Population genomics data of wheat progenitors are also available, including data for wild and domesticated emmers (AABB) [29,30] and of Aegilops tauschii (DD) [31].

Using all the above published datasets (Table 2), we constructed the "*Wheat-SnpHub-Portal*" website. The VCF files from He *et al.* [4] and Pont *et al.* [5] were downloaded from the links provided in their original papers. For datasets from Cheng et al. [6] and Wang et al. [29], the genotyping data in VCF formats were shared by the authors. For dataset from Singh *et al.* [31], raw sequence reads were downloaded from NCBI SRA under accession SRP141206 and VCF files were regenerated using scripts provided in the paper. As for dataset of Jordan et al. [28] and Avni et al. [30], we downloaded raw sequence data from NCBI SRA under SRP167848 and SRP032974, respectively. Raw reads were then trimmed using Trimmomatic [32] and

aligned to reference genomes using BWA-MEM [33]. SNPs and INDELs were identified with HaplotypeCaller module of GATK [34] and were further filtered by VariantFiltration function with the parameter "QD<2.0 || FS>60.0 || MQRankSum<−12.5 || ReadPosRankSum<− 8.0 || SOR>3.0 || MQ< 40.0 || DP >30 || DP < 3." and "QD< 2.0 || FS>200.0 || ReadPosRankSum<−20.0 || DP>30 || DP< 3", respectively. Generally, with the provided configuration data and variation files, the pre-processing step can be quickly finished, taking from ~8 minutes [5] to ~4 hours [6].

The *Wheat-SnpHub-Portal* website is designed as a portal website for providing multiple querying servers of variation databases for wheat and its progenitor species. Researchers studying wheat or wheat progenitors can easily explore multiple genomic variation datasets, as supported by SnpHub framework. The *Wheat-SnpHub-Portal* website will be updated with further released genomic variation datasets of wheat and its progenitors in the future.

Discussions

With the decreasing sequencing costs, increased numbers of samples and species will be sequenced. That will be difficult for universal and centralized databases to satisfy the versatile needs for variant analysis and querying new datasets. SnpHub can be applied to any species with an assembled genome and gene annotations. It can be instantly set up based on the VCF files. For the future population genetic studies, a SnpHub querying server can be easily set up in addition to the publication of the raw data generated, making the data to be more easily accessible by the community. SnpHub can serve as laboratory-level web server for navigating and visualizing the genomic

diversity or individual line or lineage. SnpHub can be useful for different occasions: investigators can infer trait-associated genes with population structure information and variation function annotations from specific sample sets; and breeders can access the genetic diversity at specific loci for designing new breeds. SnpHub provides a uniform server framework for easily setting up distributed servers for genotyping-queries and analysis, and can be used to build database portals such as our *Wheat-SnpHub-Portal*, extending this strategy from wheat to other important crops or other plants.

Competing interests

The authors declare that they have no competing interests.

Availability of supporting source code and requirements

Project name: SnpHub

Project home page: https://guoweilong.github.io/SnpHub/

Research Resource Identifier: RRID:SCR_018177; biotoolsID:SnpHub

Operating system(s): Linux

Programming language: R, Shell

Other requirements: R/Shiny, samtools, bcftools, seqkit, tabix

License: MIT licence

An archival copy of the github repository is available via the GigaScience database GigaDB [35].

Funding

This work has been supported by the National Natural Science Foundation of China [grant number 31701415] and the National Key Research and Development Program of China [grant number 2018YFD0100803 and 2016YFD0100801].

Authors' contributions

Method development: W.W., Z.W., W.G.; implementation: W.W., Z.W., X.L., W.G.; data preparation: Z.W.; design and testing: W.W., Z.W., Z.N., M.X., H.P., Y.Y., Q.S., W.G.; definition of research project: W.G.

Acknowledgements

We thank Prof. Yu Jiang and Prof. Lingrang Kong for sharing their processed VCF files. We thank Xiaoming Xie, Yongming Chen, Zhengzhao Yang for exploring technology, and thank Kuohai Yu for IT support.

Figures

Figure 1. Design schema of the SnpHub server. Once the files and information tables are provided as indicated in the "Prepare" step, the SnpHub server instance performs a pre-processing step for building basic database files and then runs through the Shiny framework. Users can query specific genomic regions or genes for either pre-defined or custom sample groups. SnpHub can efficiently load the raw query data from the hard disk to RAM and then performs an efficient analysis and visualization interactively.

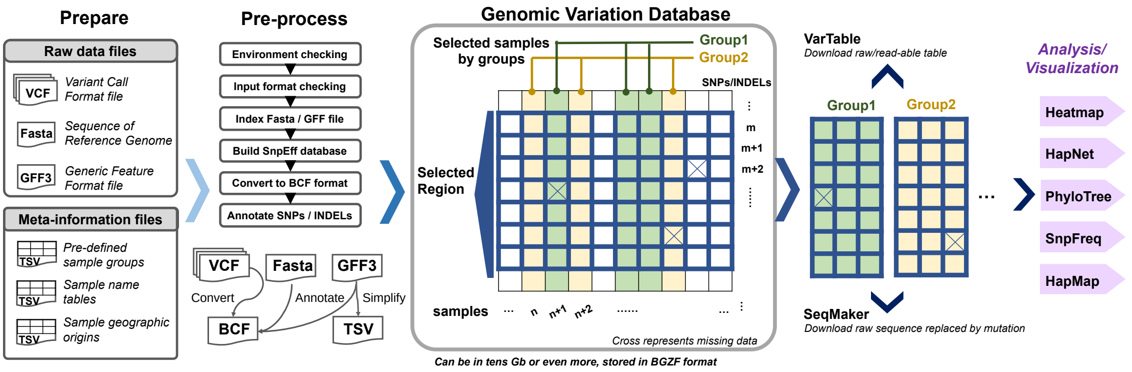

Figure 2. Analysis and visualization functions of the SnpHub server. In one SnpHub instance, each function is implemented in an independent webpage tab.

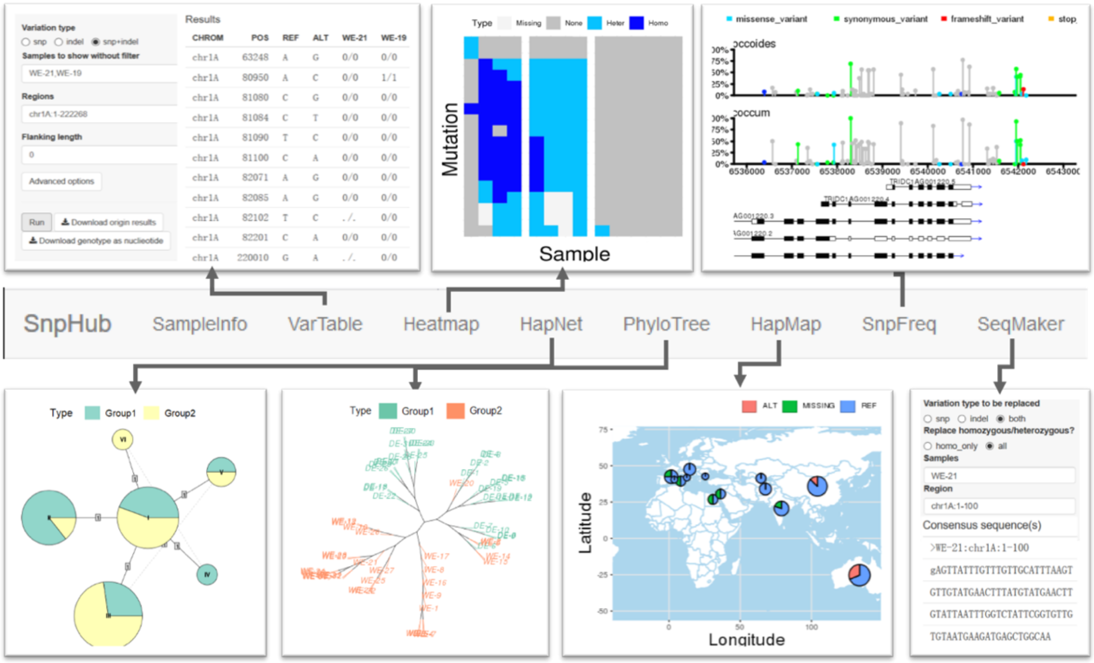

Tables

Table 1. Features supported by SnpHub, Gigwa v2, CanvasDB and JBrowse.

Features	SnpHub	Gigwa v2	CanvasDB	JBrowse
General designment	Specialized	Specialized	Specialized	Generalized
Main strengths in querying data	Query with supports of exportation, visualization and reanalysis	Query with API for external visualization	Query with filtering functions	Track-based query and visualization
Database implementation	Indexed BCF	MongoDB	MySQL	Indexed VCF
Programming language	R/Shell	Java/JavaScript	R	JavaScript/Perl
Support downstream haplotype analyze	Yes	No	No	No
Allow sample selection	Yes	Yes	Yes	No
Forms of results	Table&Figure	Table	Table	Track-based plot
Support user-defined groups	No limitation in group number	<=2 groups	No	No
Deployment difficulty for bioinformaticians	Easy	Hard	Hard	Moderate
Visualizing variations across gene structure	Yes	No	No	Yes
Visualizing samples passports geographically	Yes	No	No	No
Access to metadata	Yes, user readable	Yes, built-in	No	Yes, user readable
Accession name management strategy	Triple-name strategy	--	--	--
Retrieval consensus sequence	Yes	No	No	No

Table 2. The SnpHub instances available in *Wheat-SnpHub-Portal*. WGS, whole-genome resequencing. WEC, Whole-genome Exon-Capture. GBS, Genotyping-By-Sequencing. Disk usage refers to the size of BCF files. *Data is re-analysed from raw sequencing data.

Ploidy	Method	#Sample	Disk usage	Source
Tetraploid	WGS	35	15.0GB	Wang *et al.* 2020
Hexa-/Tetra-/Diploid	WGS	63/25/5	39.8GB	Cheng *et al.* 2019
Hexa-/Tetra-/Diploid	WEC	436/38/13	192MB	Pont *et al.* 2019
Hexaploid	WEC	1026	1.8GB	He *et al.* 2019

Hexaploid	WEC&GBS	62	2.4GB*	Jordan *et al.* 2015
Tetraploid	WEC	64	645MB	Avni *et al.* 2017
Diploid	GBS	567	234MB*	Singh *et al.* 2019

References

1. Zhou Z, Jiang Y, Wang Z, Gou Z, Lyu J, Li W, et al. Resequencing 302 wild and cultivated accessions identifies genes related to domestication and improvement in soybean. Nature Biotechnology. 2015;33:408–14.

2. Chia J-M, Song C, Bradbury PJ, Costich D, de Leon N, Doebley J, et al. Maize HapMap2 identifies extant variation from a genome in flux. Nature Genetics. 2012;44:803–7.

3. Chapman JA, Mascher M, Buluç A, Barry K, Georganas E, Session A, et al. A whole-genome shotgun approach for assembling and anchoring the hexaploid bread wheat genome. Genome Biology. 2015;16:26.

4. He F, Pasam R, Shi F, Kant S, Keeble-Gagnere G, Kay P, et al. Exome sequencing highlights the role of wild-relative introgression in shaping the adaptive landscape of the wheat genome. Nature Genetics. 2019;51:896–904.

5. Pont C, Leroy T, Seidel M, Tondelli A, Duchemin W, Armisen D, et al. Tracing the ancestry of modern bread wheats. Nature Genetics. 2019;51:905–11.

6. Cheng H, Liu J, Wen J, Nie X, Xu L, Chen N, et al. Frequent intra- and inter-species introgression shapes the landscape of genetic variation in bread wheat. Genome Biology. 2019;20:136.

7. Danecek P, Auton A, Abecasis G, Albers CA, Banks E, DePristo MA, et al. The variant call format and VCFtools. Bioinformatics. 2011;27:2156–8.

8. IC4R Project Consortium, Hao L, Zhang H, Zhang Z, Hu S, Xue Y. Information Commons for Rice (IC4R). Nucleic Acids Research. 2016;44:D1172–80.

9. Portwood JL, Woodhouse MR, Cannon EK, Gardiner JM, Harper LC, Schaeffer ML, et al. MaizeGDB 2018: the maize multi-genome genetics and genomics database. Nucleic Acids Research. 2019;47:D1146–54.

10. Mansueto L, Fuentes RR, Chebotarov D, Borja FN, Detras J, Abriol-Santos JM, et al. SNP-Seek II: A resource for allele mining and analysis of big genomic data in Oryza sativa. Current Plant Biology. Elsevier B.V.; 2016;7–8:16–25.

11. Ameur A, Bunikis I, Enroth S, Gyllensten U. CanvasDB: a local database infrastructure for analysis of targeted- and whole genome re-sequencing projects. Database. 2014;2014:bau098–bau098.

12. Sempéré G, Pétel A, Rouard M, Frouin J, Hueber Y, De Bellis F, et al. Gigwa v2-Extended and improved genotype investigator. GigaScience. 2019;8:1–9.

13. Dereeper A, Homa F, Andres G, Sempere G, Sarah G, Hueber Y, et al. SNiPlay3: a web-based application for exploration and large scale analyses of genomic variations. Nucleic Acids Research. 2015;43:W295–300.

14. Li H, Handsaker B, Wysoker A, Fennell T, Ruan J, Homer N, et al. The Sequence Alignment/Map format and SAMtools. Bioinformatics. 2009;25:2078–9.

15. Li H. A statistical framework for SNP calling, mutation discovery, association mapping and population genetical parameter estimation from sequencing data. Bioinformatics. 2011;27:2987–93.

16. Shen W, Le S, Li Y, Hu F. SeqKit: A Cross-Platform and Ultrafast Toolkit for FASTA/Q File Manipulation. Zou Q, editor. PLOS ONE. 2016;11:e0163962.
17. Li H. Tabix: Fast retrieval of sequence features from generic TAB-delimited files. Bioinformatics. 2011;27:718–9.
18. Wickham H. ggplot2. Journal of the Royal Statistical Society: Series A (Statistics in Society). Cham: Springer International Publishing; 2016.
19. Kahle D, Wickham H. ggmap: Spatial Visualization with ggplot2. The R Journal. 2013;5:144.
20. Paradis E. pegas: an R package for population genetics with an integrated-modular approach. Bioinformatics. 2010;26:419–20.
21. Knaus BJ, Grünwald NJ. vcfr: a package to manipulate and visualize variant call format data in R. Molecular Ecology Resources. 2017;17:44–53.
22. Paradis E, Schliep K. ape 5.0: an environment for modern phylogenetics and evolutionary analyses in R. Schwartz R, editor. Bioinformatics. 2019;35:526–8.
23. Cingolani P, Platts A, Wang LL, Coon M, Nguyen T, Wang L, et al. A program for annotating and predicting the effects of single nucleotide polymorphisms, SnpEff. Fly. 2012;6:80–92.
24. Huerta-Sánchez E, Jin X, Asan, Bianba Z, Peter BM, Vinckenbosch N, et al. Altitude adaptation in Tibetans caused by introgression of Denisovan-like DNA. Nature. 2014;512:194–7.
25. Guo W, Zhu P, Pellegrini M, Zhang MQ, Wang X, Ni Z. CGmapTools improves the precision of heterozygous SNV calls and supports allele-specific

methylation detection and visualization in bisulfite-sequencing data. Birol I, editor. Bioinformatics. 2018;34:381–7.

26. Buels R, Yao E, Diesh CM, Hayes RD, Munoz-Torres M, Helt G, et al. JBrowse: a dynamic web platform for genome visualization and analysis. Genome Biology. 2016;17:66.

27. Mayer KFX, Rogers J, Dole el J, Pozniak C, Eversole K, Feuillet C, et al. A chromosome-based draft sequence of the hexaploid bread wheat (*Triticum aestivum*) genome. Science. 2014;345:1251788–1251788.

28. Jordan KW, Wang S, Lun Y, Gardiner L-J, MacLachlan R, Hucl P, et al. A haplotype map of allohexaploid wheat reveals distinct patterns of selection on homoeologous genomes. Genome Biology. 2015;16:48.

29. Wang H, Yin H, Jiao C, Fang X, Wang G, Li G, et al. Sympatric speciation of wild emmer wheat driven by ecology and chromosomal rearrangements. Proceedings of the National Academy of Sciences of the United States of America. 2020; 117: 5955-5963.

30. Avni R, Nave M, Barad O, Baruch K, Twardziok SO, Gundlach H, et al. Wild emmer genome architecture and diversity elucidate wheat evolution and domestication. Science. 2017;357:93–7.

31. Singh N, Wu S, Tiwari V, Sehgal S, Raupp J, Wilson D, et al. Genomic Analysis Confirms Population Structure and Identifies Inter-Lineage Hybrids in Aegilops tauschii. Frontiers in Plant Science. 2019;10:9.

32. Bolger AM, Lohse M, Usadel B. Trimmomatic: a flexible trimmer for Illumina sequence data. Bioinformatics. 2014;30:2114–20.

33. Li H. Aligning sequence reads, clone sequences and assembly contigs with BWA-MEM. arXiv. 2013.

34. McKenna A, Hanna M, Banks E, Sivachenko A, Cibulskis K, Kernytsky A, et al. The Genome Analysis Toolkit: A MapReduce framework for analyzing next-generation DNA sequencing data. Genome Research. 2010;20:1297–303.

35. Wang W; Wang Z; Li X; Ni Z; Hu Z; Xin M; Peng H; Yao Y; Sun Q; Guo W: Supporting data for "SnpHub: an easy-to-set-up web server framework for exploring large-scale genomic variation data in the post-genomic era with applications in wheat." GigaScience Database. 2020 http://dx.doi.org/10.5524/100745
