# Supplementary material for: SnpHub: an easy-to-set-up web server framework for exploring large-scale genomic variation data in the post-genomic era with applications in wheat
Source: Gigascience. 2020 Jun 5;9(6):giaa060. doi: 10.1093/gigascience/giaa060 (PMC7274028; doi:10.1093/gigascience/giaa060)
Supplement: giaa060_Response_to_Reviewer_Comments_Original_Submission [file giaa060_response_to_reviewer_comments_original_submission.pdf]

Author's Response To Reviewer Comments

Close

Dear Editor,

We are very thankful to you and all the reviewers for your constructive comments to help improve our manuscript “SnpHub: an easy-to-set-up web server framework for exploring large-scale genomic variation data in the post-genomic era with applications in wheat” (GIGA-D-20-00003). We have considered all comments and suggestions and carefully revised the manuscript.

Generally, our modifications include the following main aspects:
1. A comparison between SnpHub and other three similar applications (Gigwa v2, CanvasDB and JBrowse) have been added, to help user decide on when to use SnpHub based on their actual needs. And new table for functional comparison (Table 1) were added in the revised manuscirpt.
2. As Wang et al. recently published new dataset on wild emmer wheat, we currently have included this dataset in Wheat-SnpHub-Portal database. The details in the Table2 (original Table 1) were also edited to be consistent with http://wheat.cau.edu.cn/Wheat_SnpHub_Portal/.
3. A new paragraph is added for explaining how data behind Wheat-SnpHub-Portal were prepared to get the VCF files.
4. We have updated SnpHub homepage and tutorial webpage, by correcting the links, fixed typos, rewrite to improve the grammars, and also have added animation illustrations for each function in forms of GIF figures.
5. We have included in SnpHub with support for integrating microarray data in hapmap format.
6. All the R packages and tools utilized in SnpHub have been cited or listed with URLs in the revised manuscript.
7. We have registered SnpHub in the bio.tools and SciCrunch.org databases, and have provided RRID and biotoolsID identifiers in the section of “Availability of supporting source code and requirements”.

The point-by-point response to comments and questions from the reviewers point-by-point are also attached.

Thank you again for all of your assistance.

Sincerely,
Weilong Guo

****
REVIEWS
****
Reviewer #1:

The authors present their software SnpHub for data exploration of VCF files. The software is a useful contribution to the crop genomics community and has brought together a number of existing tools to provide a range of features in a Shiny/R framework. The authors provide a web page with installation instructions and guides on usage. I have two main comments for the authors that I think should be addressed.

Reply: Thanks for the reviewer’s positive comments. We have substantially revised the manuscript upon these valuable suggestions.

1) Although the authors point out that similar tools such as Gigwa v2 and CanvasDB exist, the comparison seems cursory. The authors report that the main benefit of SnpHub is more efficient management of variant data, but this is not further supported. For example, the table (Table 1) showing the disk usage of the wheat data sets does not compare the potential disk usage if using other tools to build a queryable database. The manuscript may benefit from some further comparison that would allow

readers to decide on when to use SnpHub, rather than other tools, based on their specific needs.

Reply: We thank the reviewer for this beneficial suggestion. We agree that providing such a comparison will be useful for readers. We have added a new paragraph and a new table (Table 1 in revised manuscript) for comparing SnpHub with Gigwa v2, CanvasDB and JBrowse.

The corresponding revised paragraph and new table run as following:

"Advantages of SnpHub in managing variation data
SnpHub is designed as a database framework specialized for retrieving and light-weighted analysis of large genomic variation data. To provide instant responses for queries and interactive analysis, SnpHub focuses on the supports for haplotype analysis or genomic variation analysis for specific region or gene, rather than genome-wide scale analysis such as GWAS analysis. For a clear view on the advantages of SnpHub, a comprehensive comparison on their supported features is presented (Table 1) with three other popular frameworks. Both Gigwa v2 [12] and CanvasDB [11] are specialized framework for investigating genotype data, and are implemented with SQL-based database engines. The SQL-based servers generally require reload genotype information into specialized database tables, and meanwhile lost the resourceful meta-information in VCF files for describing variations. SnpHub is actually based on BCF format, which is lossless binary converted format of VCF and wildly used by bioinformaticians, and thus will save disk storage in practice. JBrowse [26] is general-purpose genome browser framework, and provide flexible visualization and querying functions, while with shortcoming in support of re-analyses. In contrast, SnpHub is designed with R/Shiny framework, providing a variety of both visualization and re-analysis functions. Moreover, as R packages and R/Shiny framework are wildly accepted by the bioinformatician communities, it would be easier for SnpHub to incorporate powerful analysis function and be extended. In general, SnpHub allowed users an alternative choice for interactively exploring the huge genomic diversity data and are more strengthen in performing light-weight re-analyses, including group-wise comparison, haplotype-related analysis, phylogenetic analysis, passport visualization, retrieving consensus sequences and generating processable tables and figures."

2) I could not access the SnpHub Wheat Portal using the provided link (http://wheat.cau.edu.cn/Wheat_SnpHub_Portal/), making it difficult to evaluate this aspect of the paper. As the wheat portal is also a resource presented by the paper, this should be available. I had the same issue after attempting to access the link on different days and using different browsers (Chrome and Firefox), though I cannot rule out that the issue was on my side.

Reply: We thank the reviewers for letting us be aware of the inaccessible of the Wheat-SnpHub-Portal. We have checked about it and found that there has been a two weeks reconstruction of campus network, which making it failed to access our new website outside the campus, while it works well within the campus. Currently, the problem is solved, and should be able to accessed globally.
Moreover, to avoid such case in the future, we have added monitoring server from proxy to keep us aware with accessible status from outside. Email feedbacks for accessing the server are welcomed in the future, and we'll try the best to keep it accessible.

Minor comments
3) The authors may want to cite all of the bioinformatics software which their tool relies on. On page 6 the authors write "Several widely used bioinformatics software programs must be pre-installed, such as SAMtools [14], bcftools [15], seqkit [16] and Tabix [17], along with several R packages". These R packages should be cited, particularly if they have been published in scientific journals as, for example, vcfR has (Knaus, Brian J., and Niklaus J. Grunwald. 2017. VCFR: a package to manipulate and visualize variant call format data in R. Molecular Ecology Resources 17(1):44-53).

Reply: We thank the reviewer for the valuable suggestions. We have gone through the manuscript and tried the best to add citations for published softwares, those SnpHub relies on, including ggplot2, ggmap, pegas, vcfR, ape, etc. For the packages or softwares has not been published in scientific journals, we also have added the URLs for accessing the source code.

4) Pg 14: The authors state "We downloaded all the above published datasets (Table 1), and then generated VCF files from raw sequencing data or utilized the published VCF files directly." Please add detail of how the VCFs were generated. If the data is meant to be used as a resource, it must be clear to users how it was generated.

Reply: We thank the reviewer for this concern. We have added a corresponding paragraph for describing

the generation process of VCF files with detailed parameters. And a sentence is added for connecting two paragraphs.
The added descriptions of VCF file generation run as follows:

“Using all the above published datasets (Table 2), we constructed up the “Wheat-SnpHub-Portal” website. The VCF files from He et al. [4] and Pont et al. [5] were downloaded from the links provided in their original papers. For datasets from Cheng et al. [6] and Wang et al. [29], the genotyping data in VCF formats were shared by the authors. For dataset from Singh et al. [31], raw sequence reads were downloaded from NCBI SRA under accession SRP141206 and VCF files were regenerated using scripts provided in the paper. As for dataset of Jordan et al. [28] and Avni et al. [30], we downloaded raw sequence data from NCBI SRA under SRP167848 and SRP032974, respectively. Raw reads were then trimmed using Trimmomatic [32] and aligned to reference genomes using BWA-MEM [33]. SNPs and INDELs were identified with HaplotypeCaller module of GATK [34] and were further filtered by VariantFiltration function with the parameter “QD<2.0 || FS>60.0 || MQRankSum<−12.5 || ReadPosRankSum<− 8.0 || SOR>3.0 || MQ< 40.0 || DP >30 || DP < 3.” and “QD< 2.0 || FS>200.0 || ReadPosRankSum<−20.0 || DP>30 || DP< 3”, respectively. Generally, with the provided configuration data and variation files, the pre-processing step can be quickly finished, taking from ~8 minutes [5] to ~4 hours [6].”

5) There are some minor errors in the web pages for snphub, so it may be worthwhile going over some of these. Two of the errors I found were as follows. I think making sure that the installation and set up go as smoothly as possible, particularly for the biologists without a programming background that SnpHub is aimed at, will be an important aspect of helping this tool get taken up by the community.
On the github page (https://github.com/esctrionsit/snphub) the authors state "Edit the setup_config.R file, make sure all the paths are correct." However the file "setup_config.R" does not exist in the github repo, instead I think the file is called "setup.R".
The github link on the top of the quick start description (https://esctrionsit.github.io/snphub_tutorial/content/Setup/quickstart.html) is broken for me.

Reply: We thank the reviewer for figuring out these inconsistence and mistakes in the document webpage, which is created due to the recent updates in documents and websites. We have corrected the links in homepages, double checked the accessibility of all links in our websites, and also have tried best to corrected the tyros and grammar mistakes in the documents.

For the issue with “setup_config.R”, the configuration file of SnpHub has been renamed from “setup_config.R” to “setup.conf”, to make it more explicit to users. The SnpHub homepage (https://guoweilong.github.io/SnpHub/) and tutorial webpage (https://esctrionsit.github.io/snphub_tutorial/) have both been updated.
The documents for “general setup” can be found at https://esctrionsit.github.io/snphub_tutorial/content/Setup/quick_deploy.html.
The documents for “quick setup with Docker” can be found at https://esctrionsit.github.io/snphub_tutorial/content/Docker/overview.html .

****
Reviewer #2:

The publication and the resource developed for has merits and provided handy tools for analysis of SNP data.
1) A major shortcoming is the analysis of the SNP array data. Can they include an interface to analyse SNP array data, where the data is usually available in .hapmap format.

Reply: We thank the reviewer for this useful suggestion. We have added the support for importing the SNP array data which are usually available in .hapmap format.
In the latest version of SnpHub, We have added an utility to convert hapmap format to VCF. Such format conversion can be done with following command.

```
python snphub/data_transfer/hapmap2vcf.py -i [input hapmap path] -o [output vcf path]
```

Then the new generated VCF files can be feed with the whole SnpHub pipelines. And we have also updated this new feature on the tutorial webpage

(https://esctrionsit.github.io/snphub_tutorial/content/QA/QA.html#inputoutput-formats).

2) I will suggest to add a video tutorial for using snpHub. Please see a recent rice galaxy paper published in Gigascience. It would greatly help breeding community, which sometime don't have expertise for such analyses.

Reply: We thank the reviewer for this suggestion. We agree with the reviewer on improving the tutorial by adding illustration videos. While considering the compatibility with our documenting system, we currently decide to add animation figures (in GIF format) to illustrate the usage of document website. We have recorded the basic usage of each function as GIFs, illustrating how to click and fill the parameters, and what would be shown in output as the examples.
Here is the link for one example:
https://esctrionsit.github.io/snphub_tutorial/content/Basic_Usage/vartable.html#demonstration

3) No bioinformatics pipeline is embedded in the resource, this could be easily incorporated to add a blast tool or annotation of wheat genome sequence.

Reply: Actually, we have included many bioinformatics analysis functions in SnpHub, including heatmap, haplotype-network analysis and construction of phylogenetic trees. For the annotation of variations, the VCF file will be annotated by SnpEff using provided GFF3 file in our pre-processing step, thus annotation information can be displayed in "VarTable", "Heatmap" and "SnpFreq" functions.
As we have described in the manuscript, Snphub is designed as a database framework specialized for management the querying for genomic variation data, with advantages in managing, supporting querying and reanalysis for SNP and indels.
Some bioinformatics functions, such as blast and query for gene functions, are supposed to be useful, and have already been implemented or integrated in general-purpose databases. These applications can be widely found, and have few connections with the design purpose of SnpHub. We would not plan to add such function in the framework of SnpHub. However, we believe it would be useful for building the comprehensive database, such as the I (http://202.194.139.32/).
Moreover, we added a clarification for describing the scope of Snphub in the revised manuscript. It runs as following:
"SnpHub is designed as a database framework specialized for retrieving and light-weighted analysis of large genomic variation data."

4) Since it is more focused to be a visualization tool, I would suggest if the variants could be visualized in wheat JBrowse.

Reply: We appreciate the reviewer providing this suggestion.
Actually, SnpHub is an independent database framework with JBrowse. And many designed functions of SnpHub are complementary with the functions of JBrowse. The implemented strategies of SnpHub and JBrowse are different. For example, JBrowse forcus on the visualization of variations through tracked view, and SnpHub forcus on data management, data retrieving and data re-analyzation together with visualization of some results.
In the revised manuscript, we have added the following descriptions for comparing SnpHub and JBrowse. And we also included JBrowse for feature comparison, as suggested the reviewer #1.

"JBrowse [26] is general-purpose genome browser framework, and provide flexible visualization and querying functions, while with shortcoming in support of re-analyses. In contrast, SnpHub is designed with R/Shiny framework, providing a variety of both visualization and re-analysis functions. Moreover, as R packages and R/Shiny framework are wildly accepted by the bioinformatician communities, it would be easier for SnpHub to incorporate powerful analysis function and be extended."

Close
