# Supplementary material for: SnpHub: an easy-to-set-up web server framework for exploring large-scale genomic variation data in the post-genomic era with applications in wheat
Source: Gigascience. 2020 Jun 5;9(6):giaa060. doi: 10.1093/gigascience/giaa060 (PMC7274028; doi:10.1093/gigascience/giaa060)
Supplement: giaa060_Reviewer_1_Report_Original_Submission — Armin Scheben -- 1/24/2020 Reviewed [file giaa060_reviewer_1_report_original_submission.pdf]

Reviewer Report

Title: SnpHub: an easy-to-set-up web server framework for exploring large-scale genomic variation data in the post-genomic era with applications in wheat

Version: Original Submission **Date:** 1/24/2020

Reviewer name: Armin Scheben

Reviewer Comments to Author:

The authors present their software SnpHub for data exploration of VCF files. The software is a useful contribution to the crop genomics community and has brought together a number of existing tools to provide a range of features in a Shiny/R framework. The authors provide a web page with installation instructions and guides on usage. I have two main comments for the authors that I think should be addressed.

1) Although the authors point out that similar tools such as Gigwa v2 and CanvasDB exist, the comparison seems cursory. The authors report that the main benefit of SnpHub is more efficient management of variant data, but this is not further supported. For example, the table (Table 1) showing the disk usage of the wheat data sets does not compare the potential disk usage if using other tools to build a queryable database. The manuscript may benefit from some further comparison that would allow readers to decide on when to use SnpHub, rather than other tools, based on their specific needs.

2) I could not access the SnpHub Wheat Portal using the provided link (http://wheat.cau.edu.cn/Wheat_SnpHub_Portal/), making it difficult to evaluate this aspect of the paper. As the wheat portal is also a resource presented by the paper, this should be available. I had the same issue after attempting to access the link on different days and using different browsers (Chrome and Firefox), though I cannot rule out that the issue was on my side.

Minor comments

3) The authors may want to cite all of the bioinformatics software which their tool relies on. On page 6 the authors write "Several widely used bioinformatics software programs must be pre-installed, such as SAMtools [14], bcftools [15], seqkit [16] and Tabix [17], along with several R packages". These R packages should be cited, particularly if they have been published in scientific journals as, for example, vcfR has (Knaus, Brian J., and Niklaus J. Grunwald. 2017. VCFR: a package to manipulate and visualize variant call format data in R. Molecular Ecology Resources 17(1):44-53).

4) Pg 14: The authors state "We downloaded all the above published datasets (Table 1), and then generated VCF files from raw sequencing data or utilized the published VCF files directly." Please add detail of how the VCFs were generated. If the data is meant to be used as a resource, it must be clear to users how it was generated.

5) There are some minor errors in the web pages for snphub, so it may be worthwhile going over some of these. Two of the errors I found were as follows. I think making sure that the installation and set up go as smoothly as possible, particularly for the biologists without a programming background that SnpHub is aimed at, will be an important aspect of helping this tool get taken up by the community.

On the github page (https://github.com/esctrionsit/snphub) the authors state "Edit the setup_config.R

file, make sure all the paths are correct." However the file "setup_config.R" does not exist in the github repo, instead I think the file is called "setup.R".
The github link on the top of the quick start description (https://esctrionsit.github.io/snphub_tutorial/content/Setup/quickstart.html) is broken for me.

Level of Interest

Please indicate how interesting you found the manuscript: Choose an item.

Quality of Written English

Please indicate the quality of language in the manuscript: Choose an item.

Declaration of Competing Interests

Please complete a declaration of competing interests, considering the following questions:

- Have you in the past five years received reimbursements, fees, funding, or salary from an organisation that may in any way gain or lose financially from the publication of this manuscript, either now or in the future?
- Do you hold any stocks or shares in an organisation that may in any way gain or lose financially from the publication of this manuscript, either now or in the future?
- Do you hold or are you currently applying for any patents relating to the content of the manuscript?
- Have you received reimbursements, fees, funding, or salary from an organization that holds or has applied for patents relating to the content of the manuscript?
- Do you have any other financial competing interests?
- Do you have any non-financial competing interests in relation to this paper?

If you can answer no to all of the above, write 'I declare that I have no competing interests' below. If your reply is yes to any, please give details below.

I declare that I have no competing interests

I agree to the open peer review policy of the journal. I understand that my name will be included on my report to the authors and, if the manuscript is accepted for publication, my named report including any attachments I upload will be posted on the website along with the authors' responses. I agree for my report to be made available under an Open Access Creative Commons CC-BY license (http://creativecommons.org/licenses/by/4.0/). I understand that any comments which I do not wish to be included in my named report can be included as confidential comments to the editors, which will not be published.

Choose an item.

To further support our reviewers, we have joined with Publons, where you can gain additional credit to further highlight your hard work (see: https://publons.com/journal/530/gigascience). On publication of this paper, your review will be automatically added to Publons, you can then choose whether or not to claim your Publons credit. I understand this statement.

Yes Choose an item.
